# Fluorination Effects on NOS Inhibitory Activity of Pyrazoles Related to Curcumin

**DOI:** 10.3390/molecules200915643

**Published:** 2015-08-28

**Authors:** Carla I. Nieto, María Pilar Cabildo, María Pilar Cornago, Dionisia Sanz, Rosa M. Claramunt, María Carmen Torralba, María Rosario Torres, José Elguero, José A. García, Ana López, Darío Acuña-Castroviejo

**Affiliations:** 1 Departamento de Química Orgánica y Bio-Orgánica, Facultad de Ciencias, Universidad Nacional de Educación a Distancia, Paseo Senda del Rey, 9, Madrid 28040, Spain; E-Mails: carla.nieto@hotmail.com (C.I.N.); pcabildo@ccia.uned.es (M.P.C.); mcornago@ccia.uned.es (M.P.C.); dsanz@ccia.uned.es (D.S.); 2 Departamento de Química Inorgánica I and CAI de Difracción de Rayos-X, Facultad de Ciencias Químicas, Universidad Complutense de Madrid (UCM), Madrid 28040, Spain; E-Mail: mrtorres@quim.ucm.es; 3 Instituto de Química Médica, Centro de Química Orgánica “Manuel Lora-Tamayo”, CSIC, Juan de la Cierva, 3, Madrid 28006, Spain; E-Mail: iqmbe17@iqm.csic.es; 4 Centro de Investigación Biomédica, Parque Tecnológico de Ciencias de la Salud, Universidad de Granada, Avda. del Conocimiento s/n, Armilla, 18100 Granada, Spain; E-Mails: jgsantos@ugr.es (J.A.G.); al653@cam.ac.uk (A.L.)

**Keywords:** NOS inhibitors, pyrazoles, tautomerism, fluorine derivatives, curcumin, crystallography, multinuclear NMR

## Abstract

A series of new (*E*)-3(5)-[β-(aryl)-ethenyl]-5(3)-phenyl-1*H*-pyrazoles bearing fluorine atoms at different positions of the aryl group have been synthesized starting from the corresponding β-diketones. All compounds have been characterized by elemental analysis, DSC as well as NMR (^1^H, ^13^C, ^19^F and ^15^N) spectroscopy in solution and in solid state. Three structures have been solved by X-ray diffraction analysis, confirming the tautomeric forms detected by solid state NMR. The *in vitro* study of their inhibitory potency and selectivity on the activity of nNOS and eNOS (calcium-calmodulin dependent) as well as iNOS (calcium-calmodulin independent) isoenzymes is presented. A qualitative structure–activity analysis allowed the establishment of a correlation between the presence/absence of different substituents with the inhibition data proving that fluorine groups enhance the biological activity. (*E*)-3(5)-[β-(3-Fluoro-4-hydroxyphenyl)-ethenyl]-5(3)-phenyl-1*H*-pyrazole (**13**), is the best inhibitor of iNOS, being also more selective towards the other two isoforms.

## 1. Introduction

We have been working for the last years on two different topics: (i) nitric oxide synthase (NOS) inhibitors [1,2,3], and (ii) curcumin and curcuminoid pyrazoles [4,5,6,7]. This prompted us to study the effects of new curcuminoid pyrazoles on the three NOS isoforms and analyze them by structure–activity relationships hoping to find potent/isoform specific NOS inhibitors.

Nitric oxide (NO^●^) is a free radical that serves as a multifunctional messenger affecting multiple aspects of mammalian physiology. Nitric oxide synthase (NOS) catalyzes the formation of NO^●^ from the terminal guanidino nitrogen of L-arginine. There are several homologous but separate NOS genes in mammals. Endothelial NOS (eNOS) and neuronal NOS (nNOS) enzymes are produced constitutively and they are Ca^2+^-calmodulin dependent [8]. Macrophage NOS is expressed under basal conditions and does not depend on calcium for its activity; it is an inducible NOS isoform (iNOS), which is a component of the host’s resistance to infection. The use in medicinal chemistry of different inhibitors of the NOS isoforms (nNOS, eNOS, iNOS, and mitochondrial NOS or mtNOS [9,10]) is an important goal; several families have been explored: guanidines, citrullines, amidines, isothioureas [11,12], imidazoles [13,14], benzoxazolinones [15,16] and 1*H*-indazoles [1,3,17,18,19]. More recently 4,5-dihydropyrazoles have been reported as nNOS/iNOS selective inhibitors [20]. The most relevant contributions to this topic are a series of publications that use azines as selective nNOS inhibitors [21,22,23,24,25,26].

**Figure 1:**
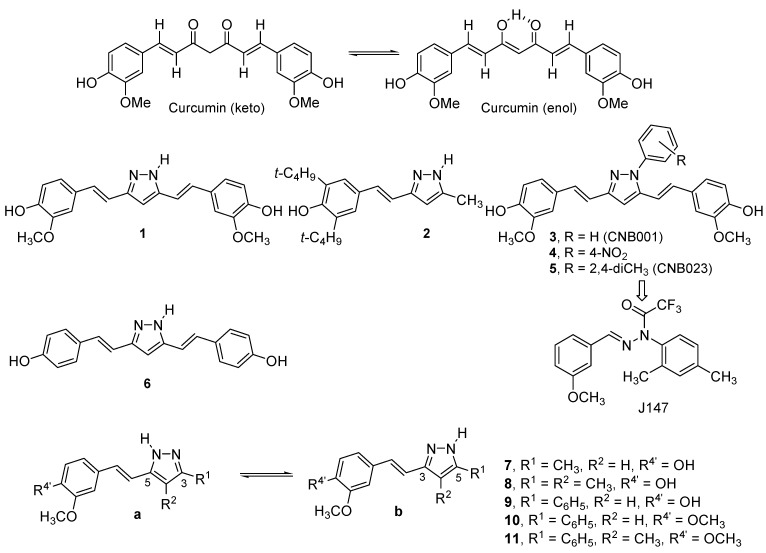
Curcuminoid pyrazoles, from the literature **1**–**6** and reported in our previous works **7**–**11**, with the definition of tautomers **a** (5-styryl) and **b** (3-styryl).

The rediscovery of the biological properties of curcumin has led to an impressive number of publications [27,28,29,30,31,32,33,34,35,36,37,38,39]. Prominent among these compounds are curcuminoid pyrazoles. Besides our own contributions [5,6], a large number of papers have been devoted to them due to their interesting properties (Figure 1).

Already in 1991, Flynn *et al.* prepared pyrazole **1** from curcumin, which is still one of the most significant compounds of this family, as well as compound **2**, and described their 5-lipooxygenase and cyclooxygenase inhibitory properties [40]. Subsequent modification did not improve **1** but other pharmacological properties were discovered, including inhibitor of endothelial cell proliferation [41] and cytotoxicity [42,43], as well as antioxidant and anti-inflammatory activity [44]. Schubert *et al.* made an important advance in preparing *N*-substituted pyrazole derivatives such as **3** (aimed at treating Alzheimer’s and Parkinson diseases), known as CNB001 [45,46,47], and **5** (CNB023) [48] and other authors prepared **4** that binds to the amyloid β peptide [49]. Compound **6** is an antiplatelet inhibitor [50]. Our previous works correspond to compound **1** and to pyrazoles **7**–**11** (Figure 1) [5,6].

In most papers dealing with curcumin, its relationship with iNOS is cited [51,52,53,54,55,56,57,58,59,60,61,62]. However, there are very few papers where curcumin is associated with the nNOS [63,64].

This is the first time in which the effects of curcuminoids on the three NOS isoforms have been simultaneously examined. Of all the literature pyrazoles related to curcumin [39,40,41,42,43,44,45,46,47,48,49], none report NOS assays; only the paper concerning J147, a non-pyrazole neurotrophic drug with iNOS properties, describes **5** (CNB023), a precursor of J147 [48].

In the present publication, we will report the unpublished biological results concerning the pyrazole series, **7**–**11**, and the synthesis, structure and biological evaluation of a new series of pyrazoles bearing fluorine atoms, **12**–**16**, designed to improve their physicochemical and medicinal properties [65].

## 2. Results and Discussion

### 2.1. Synthesis and Characterization by NMR Spectroscopy

**Figure 2:**
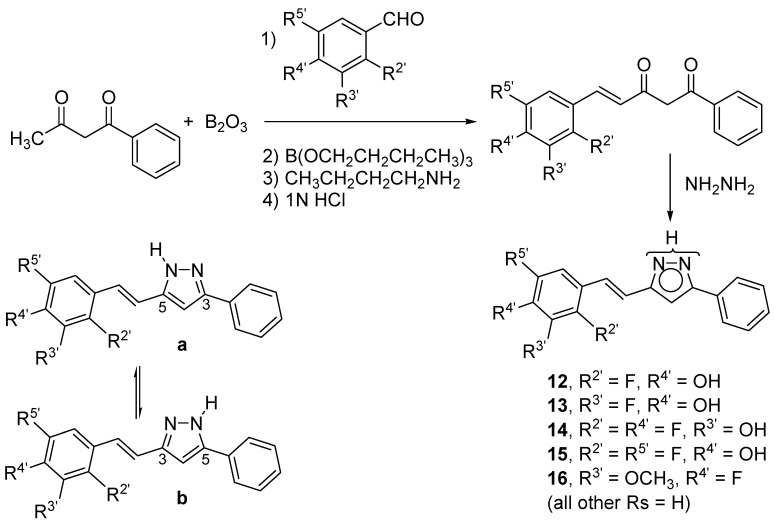
Synthetic scheme used to prepare pyrazoles **12**–**16**, that can exist in two tautomeric forms **a** and **b**.

All the compounds discussed in this work were prepared by reaction of hydrazine with the corresponding β-diketones [66,67,68]. With the exception of curcumin, which is commercially available, and was used after purification by crystallization from ethanol-water, the β-diketones were obtained as described elsewhere, according to the scheme depicted in Figure 2 [4,7]. The synthesis and characterization of pyrazoles **1** and **7**–**11**, have been previously described by us [5,6]. Full characterization of pyrazoles **12**–**16** is reported in the experimental section.

Concerning tautomerism, in the case of **1**, both tautomers are identical. In solution, either in DMSO-*d*_6_ or HMPA-*d*_18_, pyrazoles **7**–**10** proved to exist as a mixture of both forms **a** and **b** in different ratios: **7a**/**7b**, 50:50 (HMPA); **8a**/**8b**, 65:35 (HMPA); **9a**/**9b**, 64:36 (DMSO) and **10a**/**10b**, 60:40 (DMSO). For **11**, a large predominance of **11a** (DMSO) and only this form (HMPA) was established [5].

The structures of the new pyrazoles **12**–**16** have been established by ^1^H, ^13^C, ^15^N and ^19^F-NMR in solution and in solid state. All chemical shift and coupling constant data are given in the Supplementary files. The assignments of the signals to the different nuclei are based on: (i) homonuclear and heteronuclear 2D experiments; (ii) the auto-consistency of the coupling constants values; and (iii) the comparison with other *NH*-pyrazoles where tautomerization is blocked [5,69]. From our solution studies in DMSO-*d*_6_, we conclude that both tautomers coexist: **12a**/**12b**, 67:33; **13a**/**13b**, 60:40; **14a**/**14b**, 54:46; **15a**/**15b**, 60:40 and **16a**/**16b**, 57:43. In two cases (**13** and **16**), we have also used HMPA-*d*_18_, a solvent with a hydrogen-bond basicity larger than that of DMSO-*d*_6_, resulting in an 8% population increase of tautomer **a** (see Supplementary Files for ^1^H-NMR and Table 1 for ^19^F-NMR data). Using this 8% correction, we have prepared a list of **a**/**b** proportion for all pyrazoles **7**–**16** (Table 2).

As in our previous studies, ratios have been determined by integration of the ^1^H-NMR signal intensities (see Supplementary Files). Moreover, in the present paper tautomers populations have been corroborated by integration of the ^19^F-NMR signal intensities given in Table 1.

Solid state NMR spectral data indicate the presence of only one form for pyrazoles **12**–**16**, the one corresponding to the major tautomer **a** detected in solution, and also in agreement with the X-ray crystallography determination of the structures of **12**, **13** and **16** that will be discussed in the next section. Attention should be paid to the upfield effect observed on the ^19^F-NMR chemical shifts on going from solution to solid phase.

**Table 1:** ^19^F-NMR in solution and solid state of pyrazole derivatives (chemical shifts δ in ppm, *J* coupling constants in Hz).

Compound	Solvent (Temp)	F2′	F3′	F4′	F5′
**12**	DMSO-*d*_6_	−116.1 (67%)			
	295 K	−116.8 (33%)			
	MAS 300 K	−107.7			
**13**			−136.0 (60%)		
	DMSO-*d*_6_		^3^*J* = ^4^*J* = 10.8		
	295 K		−136.2 (40%)		
			^3^*J* = ^4^*J* = 10.2		
	DMSO-*d*_6_		−136.0		
	360 K				
	HMPA-*d*_18_		−137.3 (67%)		
	295 K		−137.6 (33%)		
	MAS 300 K		−129.2		
**14**	DMSO-*d*_6_	−136.4 (54%)		−132.1 (54%)	
	295 K	−137.0 (46%)		−132.8 (46%)	
	MAS 300 K	−130.7		−126.8	
**15**	DMSO-*d*_6_	−121.3 (60%)			−140.6
	295 K	−122.1 (40%)			
	MAS 300 K	−114.2			−134.8
**16**	DMSO-*d*_6_			−135.4 (57%)	
	295 K			−136.3 (43%)	
	HMPA-*d*_18_			−136.9 (65%)	
	295 K			−137.8 (35%)	
	CDCl_3_ 295 K			−135.5	
	MAS 300 K			−132.6	

**Table 2:** Proportion of tautomer **a** (**b** = 100 − **a**) for pyrazoles **7** to **16** in DMSO-*d*_6_. 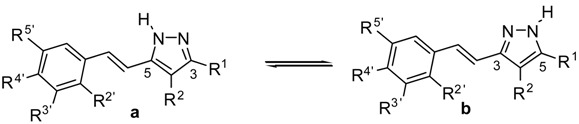

Compound	7	8	9	10	11	12	13	14	15	16
% **a**	42 ^[a]^	57 ^[b]^	64	60	85 ^[c]^	67	60	54	60	57

^[a]^ 50% in HMPA-*d*_18_; ^[b]^ 65% in HMPA-*d*_18_; ^[c]^ large excess in HMPA-*d*_18_.

**Figure 3:**
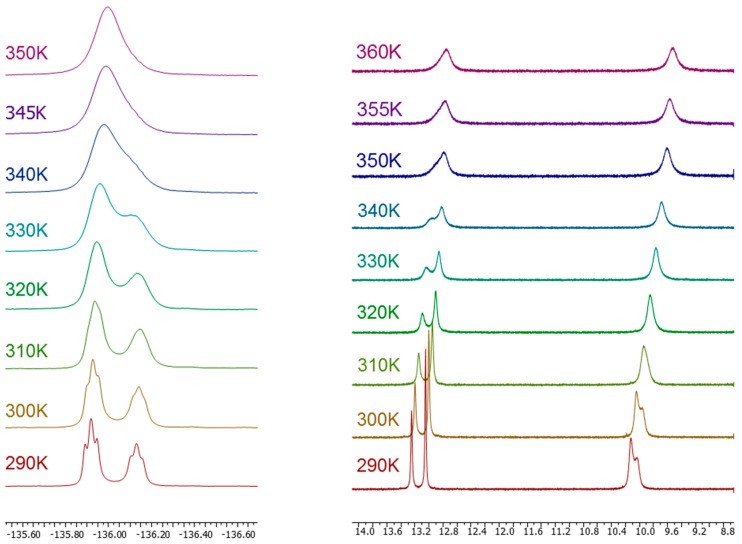
^19^F-NMR spectra (**left**) and ^1^H-NMR spectra (**right**) of compound **13** in DMSO-*d*_6_ (11 mg in 0.5 mL) in the temperature range of 290–360 K.

We have illustrated the spectra obtained in ^19^F-NMR, with the example depicted in Figure 3, which corresponds to compound (*E*)-3(5)-[β-(3-fluoro-4-hydroxyphenyl)-ethenyl]-5(3)-phenyl-1*H*-pyrazole (**13**), concentration 0.078 M, in the temperature range of 290–360 K. The dynamic NMR experiment has permitted us to estimate the barrier separating both tautomers to be 71 kJ·mol^−1^, by using the Eyring equation: ΔG_T_^‡^(kJ·mol^−1^) = 19.12 × T_c_ (10.32 + logT_c_/*k*_c_) (*k*_c_ = Δν_max_ × π/$\sqrt{2}$), a valid approach for systems in which the populations of the two sites are equal [70] using the following data: T_c_ = 350 K, Δν_max_ = 79.8, **13a**/**13b** ratio of 60:40 and *k*_c_ (s^−1^) = 177. A close value of 69 kJ·mol^−1^ was found for (*E*)-3(5)-[β-(4-fluoro-3-methoxyphenyl)-ethenyl]-5(3)-phenyl-1*H*-pyrazole (**16**) when performing a similar experiment.

### 2.2. Crystal and Molecular Structures

Suitable single crystals for X-ray diffraction studies were obtained for **12** from ethyl acetate-hexane, for **13** from dichloromethane-ethanol-hexane and for **16** from dichloromethane-hexane. In all cases, the crystallized molecule corresponds to the **a** tautomeric form, in agreement with solid NMR data.

Compounds **12** and **13** are isostructural and crystallize in the monoclinic *P*2_1_/*n* space group. For this reason, only the former will be described in detail since the results can be extended to the latter.

**Figure 4:**
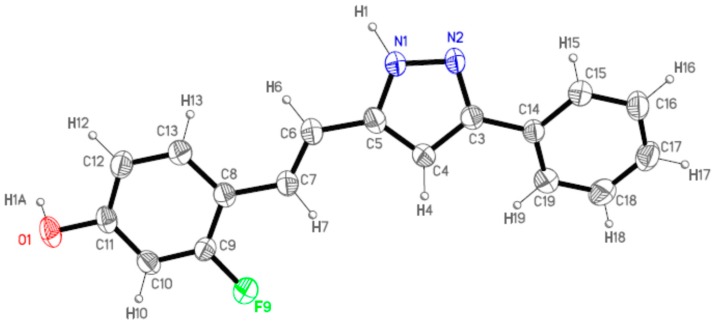
ORTEP (Oak Ridge Thermal Ellipsoid Plot) (40% ellipsoid probability) of **12** showing the labeling of the asymmetric unit.

Figure 4 shows the labeling of the asymmetric unit of **12** that corresponds to one single molecule. This molecule is not completely planar due to the twist of the phenyl ring with respect to the pyrazole moiety, the dihedral angle between them is 25.4(2)°. Excluding this phenyl ring, the rest of the atoms slightly deviated from the mean molecular plane. This fact suggests an extended electronic delocalization over this part of the molecule.

**Figure 5:**
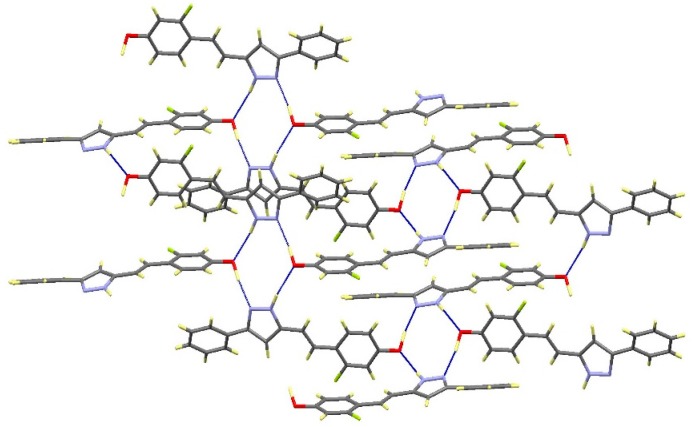
View of the packing for **12** showing the tetrameric units.

Each molecule forms intermolecular hydrogen bonds with the adjacent molecules leading to the formation of tetramers as can be shown in Figure 5 and Table 3. Every pyrazole nitrogen atom is asymmetrically hydrogen bonded to the phenol group of a different molecule, N1-H1···O1#1 and N2···H1A-O1#2 (See footnote ^[a]^ in Table 3). The tetramer is closed by the equivalent interactions of the latter with the nitrogen atoms on the centrosymmetric molecule to the initial one. The cycle so formed consists of N1···O1#1···N2#3-N1#3···O1#2···N2 (#3: 1 − x, 1 − y, 1 − z). Additionally, each molecule participates in a second tetrameric unit through its phenol group spreading out the intermolecular contacts in a bidimensional way (Figure 5).

**Table 3:** Hydrogen bonds (Å and °) for compounds **12**, **13** and **16**.

Comp	D-H···A ^[a]^	Symmetry Operations	d(D-H)	d(H···A)	d(D···A)	<(DHA)
**12**	N1-H1···O1#1	#1 –x + 1/2, y + 1/2, −z + 3/2	0.98	1.98	2.899(2)	154.1
	N2···H1A-O1#2	#2 x + 1/2, −y + 1/2, z − 1/2	1.01	1.72	2.711(2)	164.3
**13**	N1-H1···O1#1	#1 – x + 3/2, y + 1/2, −z + 1/2	1.02	1.86	2.871(3)	171.1
	N2···H1A-O1#2	#2 x − 1/2, −y + 3/2, z + 1/2	1.08	1.69	2.736(3)	161.8
**16**	N1-H1···N2#1	#1 − x, −y + 2, −z + 1	1.02	1.92	2. 852(2)	150.2

^[a]^ #1, #2 correspond to symmetry transformations used to generate equivalent atoms.

**Figure 6:**
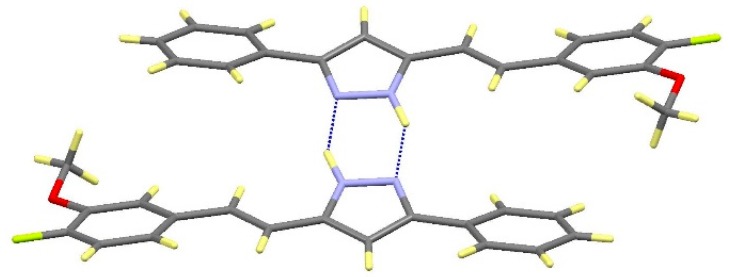
View of the dimer in **16**.

Compound **16** crystallizes in the monoclinic *P*2_1_/*c* space group. The asymmetric unit also corresponds to one molecule with a labeling scheme analogous to that shown in Figure 4 for compound **12**, but differently from **12**, both substituents on the pyrazole are twisted relative to this ring suggesting that the electronic delocalization is not extended (See Supplementary Files). Each molecule interacts with the adjacent centrosymmetric one forming dimers linked by symmetric hydrogen bonds between the pyrazole nitrogen atoms, N1-H1···N2#1 (Figure 6). No significant additional contacts have been observed. The presence of the methoxy group in **16** limits the chances of interactions leading to the formation of only isolated dimers instead of the extended network found in compounds **12** and **13**.

### 2.3. NOS Inhibitory Activity

NOS activity was measured following the conversion of l-^3^*H*-arginine to l-^3^*H*-citrulline according to the Bredt *et al.* [71] protocol and full details are given in the Experimental Section. The NOS inhibitory activities of the whole set of pyrazoles (**1**, **7**–**16**) are gathered in Table 4.

In preliminary experiments, we found that compounds **12**–**16** of the second series were much more active against NOS isoforms than the first ones. Therefore, they were assayed at concentrations enough to produce NOS inhibition similar to compounds **7**–**11**. As can be seen, 50 μM of **12**–**16** yields an inhibition of NOS isoforms comparable to 1 mM of **1**, **7**–**11**. This result means that pyrazoles **12**–**16** are about 20-fold more potent than pyrazoles **1**, **7**–**11**.

A qualitative structure–activity analysis of the data points out that, for the first series, it comes as a surprise that while curcumin and the curcuminoids are predominantly iNOS and seldom nNOS inhibitors, the curcuminoid pyrazole **1** shows the three activities in comparable amounts being predominantly an eNOS inhibitor. A 4-methyl group decreases the potency when there is a 3(5)-methyl group (compare **7** and **8**) but increases it when there is a 3(5)-phenyl group (compare **10** and **11**). Concerning potency, the best compounds are the derivatives **9** and **7** both showing about 84% inhibition but towards different isoforms (nNOS *vs.* eNOS) followed by **1** (77% towards eNOS).

For pyrazoles **12**–**16**, all of them 3(5)-phenyl derivatives, it appears that concerning nNOS the best compound is **12** but it is equally as potent as an iNOS inhibitor, with the remaining compounds being less active. Compound **13** is not only a better iNOS inhibitor, but is also more selective towards the other two isoforms than **12**. Finally, against eNOS all show similar, but weak, activity.

When comparing our results with those obtained for two standard NOS inhibitors, 7-nitro-1*H*-indazole and 4,5,6,7-tetrafluoro-1*H*-indazole [3], but under different conditions (50 μM *vs.* 1 mM), our curcuminoid pyrazoles are more potent and present different selectivities (Table 4). If compound **9** is similar in selectivity (nNOS) to 4,5,6,7-tetrafluoro-1*H*-indazole, compound **13** is higher in potency (iNOS) to 7-nitro-1*H*-indazole, but much more selective.

**Table 4:** Percentage of inhibition of nNOS, iNOS and eNOS activities in the presence of the tested curcuminoid pyrazoles **1**, **7**–**11** using 1 mM/EtOH and curcuminoid pyrazoles **12**–**16** using 50 μM/DMSO, with the most interesting values in bold. Both series compared to control (0% inhibition). Experimental data represent the means ± S.E.M. of three independent experiments (*n* = 3) each one performed in triplicate. 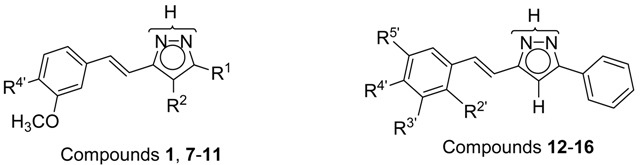

Compound	R ^1^		R ^2^	R ^4′^	nNOS	iNOS	eNOS	Predominant
**1**	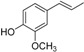		H	OH	51.2 ± 1.2	65.3 ± 2.1	77.4 ± 0.1	eNOS
**7**	CH_3_		H	OH	61.7 ± 1.6	45.6 ± 1.8	84.1 ± 0.7	eNOS
**8**	CH_3_		CH_3_	OH	29.9 ± 0.7	46.4 ± 3.2	19.2 ± 1.8	iNOS
**9**	C_6_H_5_		H	OH	84.9 ± 0.1	63.5 ± 1.9	60.3 ± 1.5	nNOS
**10**	C_6_H_5_		H	OCH_3_	20.0 ± 0.2	48.0 ± 0.9	23.4 ± 2.9	iNOS
**11**	C_6_H_5_		CH_3_	OCH_3_	19.0 ± 0.6	62.1 ± 1.2	40.6 ± 2.2	iNOS
**Compound**	**R ^2′^**	**R ^3′^**	**R ^4′^**	**R ^5′^**	**nNOS**	**iNOS**	**eNOS**	**Predominant**
**12**	F	H	OH	H	65.9 ± 6.2	65.8 ± 1.2	37.6 ± 1.8	n/iNOS
**13**	H	F	OH	H	36.8 ± 1.5	83.7 ± 6.3	37.4 ± 2.3	iNOS
**14**	F	OH	F	H	24.1 ± 2.3	33.6 ± 4.7	40.3 ± 1.6	eNOS
**15**	F	H	OH	F	38.9 ± 1.4	36.3 ± 1.2	44.2 ± 0.0	eNOS
**16**	H	OCH_3_	F	H	21.7 ± 0.3	46.0 ± 5.4	39.3 ± 3.5	iNOS
1*H*-indazoles ^[a]^								
7-nitro [3]	----				88.0	84.0	----	n/iNOS
4,5,6,7-tetrafluoro [3]	----				98.7	43.3	67.5	nNOS

^[a]^ 1 mM.

In an attempt to go further in the understanding of the effect of the fluorine atom position on the inhibitory activity, a statistical analysis of the results was carried out using a Free–Wilson matrix [72,73,74,75,76] (see Supplementary Files) differentiating the fluorine atoms according to their position. Considering that compounds **1**, **7**–**11** of the first series are 20 times less potent than those of the second series, we have divided their percentages of inhibition by 20 and have reported the normalized values together with those of the second series of pyrazoles **12**–**16**, in Table 5.

**Figure 7:**
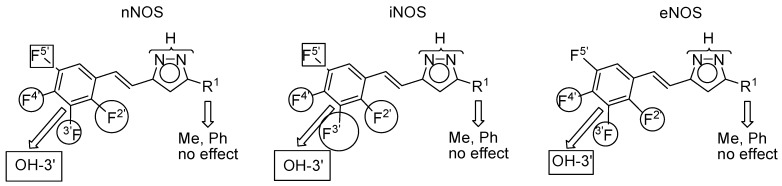
Circles, increase the activity; rectangles, decrease the activity. The size represents the importance of the effect.

The hydroxy group at position 3′ significantly lowers the potency towards all isoforms, in the order iNOS > nNOS >> eNOS. A fluorine atom at position 5′ also decreases the potency *vs.* nNOS and iNOS but to a lesser degree, and has almost no effect on the eNOS. Fluorine atoms at positions 2′, 3′ and 4′ increase the inhibitory activity *vs.* the three isoforms. The effect is usually larger on the iNOS (66, 84, 46) than on the other isoforms, contributing to the selectivity. With respect to the nNOS, the most discriminating groups are, F2′ (largest, 66) and F4′ (lowest, 22), but the position of the fluorine atom seems to have no effect (38, 37, 39) on the eNOS. We have graphically represented these conclusions in Figure 7.

**Table 5:** NOS values for compounds **1**, **7**–**16** and regression results in percentage units.

Compound	nNOS	iNOS	eNOS
**1**	2.560	3.265	3.870
**7**	3.085	2.280	4.205
**8**	1.495	2.320	0.960
**9**	4.245	3.175	3.015
**10**	1.000	2.400	1.170
**11**	0.950	3.105	2.030
**12**	65.9	65.8	37.6
**13**	36.8	83.7	37.4
**14**	24.1	33.6	40.3
**15**	38.9	36.3	44.2
**16**	21.7	46.0	39.3
F2′	66	66	38
F3′	37	84	37
F4′	22	46	39
F5′	−27	−30	7
OH-3′	−64	−78	−37
*n*	11	11	11
R^2^	0.995	0.997	0.994

## 3. Experimental Section

### 3.1. General

All chemicals cited in the synthetic procedures are commercial compounds. Melting points were determined by DSC with a SEIKO DSC 220 C connected to a model SSC5200H disk station. Thermograms (sample size 0.003–0.005 g) were recorded with a scan rate of 5.0 °C. Column chromatography was performed on silicagel (Merck 60, 70–230 mesh) and elemental analyses using a Perkin-Elmer 240 apparatus (PerkinElmer, Waltham, MA, USA).

**Figure 8:**
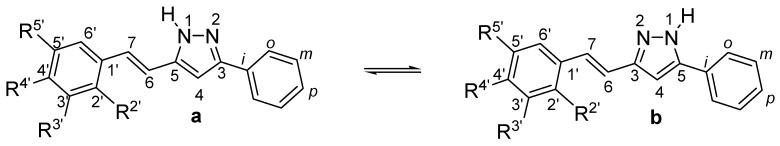
Atom numbering for NMR assignments.

The nomenclature used in the text and in the experimental is not in accordance with IUPAC rules where, for all of the compounds with phenolic hydroxyl groups, the phenol system has the highest priority; however, using IUPAC nomenclature here would be at the expense of comparability and clarity. For instance, compound **12** would be (*E*)-3-fluoro-4-(2-(3-phenyl-1*H*-pyrazol-5-yl)vinyl)phenol under IUPAC rules, rather than (*E*)-3(5)-[β-(2-fluoro-4-hydroxyphenyl)-ethenyl]-5(3)-phenyl-1*H*-pyrazole. Having decided to prioritize comparability over correct nomenclature, we have named all of the compounds as pyrazole derivatives (Figure 8).

### 3.2. NMR Parameters

Solution NMR spectra were recorded on a Bruker DRX 400 (9.4 Tesla, 400.13 MHz for ^1^H, 100.62 MHz for ^13^C, 40.54 MHz for ^15^N and 376.50 MHz for ^19^F, (Bruker Española S.A., Madrid, Spain) spectrometer with a 5-mm inverse-detection H-X probe equipped with a z-gradient coil (^1^H, ^13^C, ^15^N) and with a QNP 5 mm probe (^19^F), at 295 K. Chemical shifts (δ in ppm) are given from internal solvent, DMSO-*d*_6_ 2.49 for ^1^H and 39.5 for ^13^C, HMPA-*d*_18_ 2.51 to the upfield multiplet for ^1^H and 35.8 for ^13^C. External references were used for ^15^N and ^19^F, nitromethane and CFCl_3_, respectively. Coupling constants (*J* in Hz) are accurate to ±0.2 Hz for ^1^H, ±0.8 Hz for ^19^F and ±0.6 Hz for ^13^C. Typical parameters for ^1^H-NMR spectra were spectral width 6500 Hz and pulse width 7.5 μs at an attenuation level of 0 dB. Typical parameters for ^19^F-NMR spectra were spectral width 55 kHz, pulse width 13.75 μs at an attenuation level of −6 dB and relaxation delay 1 s. Typical parameters for ^13^C-NMR spectra were spectral width 21 kHz, pulse width 10.6 µs at an attenuation level of −6 dB and relaxation delay 2 s; WALTZ-16 was used for broadband proton decoupling; the FIDs were multiplied by an exponential weighting (lb = 2 Hz) before Fourier transformation. 2D (^1^H-^13^C) gs-HMQC and (^1^H-^13^C) gs-HMBC were acquired and processed using standard Bruker NMR software and in non-phase-sensitive mode [77]. Gradient selection was achieved through a 5% sine truncated shaped pulse gradient of 1 ms. Selected parameters for (^1^H-^13^C) gs-HMQC and gs-HMBC spectra were spectral width 3500 Hz for ^1^H and 20.5 kHz for ^13^C, 1024 × 256 data set, number of scans 2 (gs-HMQC) or 4 (gs-HMBC) and relaxation delay 1 s. The FIDs were processed using zero filling in the *F*1 domain and a sine-bell window function in both dimensions was applied prior to Fourier transformation. In the gs-HMQC experiments GARP modulation of ^13^C was used for decoupling. Selected parameters for (^1^H-^15^N) gs-HMQC spectra were spectral width 6500 Hz for ^1^H and 12.5 kHz for ^15^N, 1024 × 256 data set, number of scans 4, relaxation delay 1 s. The FIDs were processed using zero filling in the *F*_1_ domain and a sine-bell window function in both dimensions was applied prior to Fourier transformation.

Variable-temperature experiments (DNMR) were recorded with the same spectrometer with a QNP 5 mm probe and a Bruker BVT 3000 temperature unit was used to control the temperature of the cooling gas stream and an exchanger to achieve low temperatures.

Solid-state ^13^C (100.73 MHz) and ^15^N (40.60 MHz) CPMAS NMR spectra have been obtained on a Bruker WB 400 spectrometer (Bruker Española S.A.) at 300 K using a 4 mm DVT probehead. Samples were carefully packed in a 4-mm diameter cylindrical zirconia rotor (Bruker Española S.A.) with Kel-F end-caps. Operating conditions involved 3.2 μs 90° ^1^H pulses and decoupling field strength of 86.2 kHz by TPPM sequence. ^13^C spectra were originally referenced to a glycine sample and then the chemical shifts were recalculated to the Me_4_Si (for the carbonyl atom (glycine) δ = 176.1 ppm) and ^15^N spectra to ^15^NH_4_Cl and then converted to nitromethane scale using the relationship: δ ^15^N (nitromethane) = δ ^15^N (ammonium chloride) − 338.1 ppm. Typical acquisition parameters for ^13^C-CPMAS were: spectral width, 40 kHz; recycle delay, 30–100 s; acquisition time, 30 ms; contact time, 5 ms; and spin rate, 12 kHz. In order to distinguish protonated and unprotonated carbon atoms, the NQS (Non-Quaternary Suppression) experiment by conventional cross-polarization was recorded; before the acquisition the decoupler is switched off for a very short time of 25 μs [78,79,80]. For ^15^N, CPMAS were: spectral width, 40 kHz; recycle delay, 30–100 s; acquisition time, 35 ms; contact time, 6 ms; and spin rate, 6 kHz.

Solid-state ^19^F (376.94 MHz) NMR spectra have been obtained on a Bruker WB 400 spectrometer (Bruker Española S.A.) using a MAS DVT BL2.5 X/F/H double resonance probehead. Samples were carefully packed in 2.5 mm diameter cylindrical zirconia rotors with Kel-F end-caps. Samples were spun at the magic angle at rates of 25 kHz and the experiments were carried out at ambient probe temperature.

The typical acquisition parameters ^19^F{^1^H} MAS were: spectral width, 75 kHz; recycle delay, 10 s; pulse width, 2.5 μs and proton decoupling field strength of 100 kHz by SPINAL-64 sequence; recycle delay, 10 s; acquisition time, 25 ms; 128 scans; and spin rate, 25 kHz.

The ^19^F spectra were referenced to ammonium trifluoroacetate sample and then the chemical shifts were recalculated to the CFCl_3_ (δCF_3_CO_2_^−^NH_4_^+^ = −72.0 ppm).

### 3.3. General Procedure for the Preparation of Pyrazole Derivatives (***12**–**16***)

Compounds **12**–**16** were prepared by reacting the corresponding β-diketones [4,7] (1 mmol) with hydrazine hydrate 98% (1.5 mmol) in acetic acid (5 mL). After heating at reflux for 2 h the reaction mixture was poured into water. The precipitate was filtered off, washed with water and dried. The solid was purified by column chromatography using ethyl acetate/hexane as eluent or by crystallization.

*(E)-3(5)-[β-(2-Fluoro-4-hydroxyphenyl)-ethenyl]-5(3)-phenyl-1H-pyrazole* (**12**). Compound **12** was prepared from (*E*)-5-(2-fluoro-4-hydroxyphenyl)-1-phenylpent-4-ene-1,3-dione. The compound was purified by column chromatography using ethyl acetate/hexane as eluent (7:3). Yellow crystals were obtained after recrystallization from EtOH/H_2_O. Yield: (290 mg, 82%); mp: 217.3 °C; ^1^H-NMR: (400.13 MHz, DMSO-*d_6_*) δ = 6.60 (^3^*J_F_* = 12.8, ^4^*J*_H5′_ = 2.4, H_3′_), 6.65 (^3^*J*_H6′_ = 8.5, ^4^*J*_H3′_ = 2.4, H_5′_), 6.91 (H_4_), 6.99 (^3^*J*_H7_ = 16.9, H_6_), 7.17 (^3^*J*_H6_ = 16.9, H_7_), 7.30 (H*p*), 7.41 (H*m*), 7.53 (m, H_6′_), 7.80 (H*o*), 10.12 (OH), 13.06 (67%, NH), 13.23 (33%, NH); ^13^C-NMR: (100.62 MHz, DMSO-*d*_6_) δ = 99.3 (33% C_4_), 100.0 (67% C_4_), 102.8 (^2^*J*_F_ = 24.3, C_3′_), 112.3 (C_5′_), 114.9 (C_6_), 122.0 (C_7_), 122.1 (C_1′_), 125.1 (C*o*), 127.4 (C*p*), 128.2 (C_6′_), 128.7 (C*m*), 133.6 (C*i*), 142.3 (67% C_5_), 142.9 (33% C_5_), 150.9 (67%, C_3_), 151.0 (33%, C_3_), 158.7 (C_4′_), 160.5 (^1^*J*_F_ = 247.3, C_2′_); ^19^F-NMR: (376.50 MHz, DMSO-*d*_6_) δ = −116.1 (67%), −116.8 (33%); ^13^C-NMR: (100.73 MHz, CPMAS) δ = 96.9 (C_4_), 108.0 (C_3′_), 113.6 (C_5′_), 117.9 (C_6_, C_1′_), 122.0 (C_7_), 124.3 (C*o*), 126.5 (C*m*, C*p*), 128.5 (C_6__′_), 130.2 (C*i*), 144.0 (C_5_), 152.2 (C_3_), 156.2 (C_4__′_), 161.4/158.8 (C_2__′_); ^15^N-NMR: (40.60 MHz, CPMAS) δ = −111.9 (N_2_), −177.2 (N_1_); ^19^F-NMR: (376.94 MHz, MAS) δ = −107.7. Anal. calcd. for C_17_H_13_FN_2_O: C 72.85, H 4.67, N 9.99. Found: C 72.31, H 4.58, N 9.96.

*(E)-3(5)-[β-(3-Fluoro-4-hydroxyphenyl)-ethenyl]-5(3)-phenyl-1H-pyrazole* (**13**). Compound **13** was prepared from (*E*)-5-(3-fluoro-4-hydroxyphenyl)-1-phenylpent-4-ene-1,3-dione.The compound was obtained as yellow crystals after recrystallization from EtOH/CH_2_Cl_2_/hexane. Yield: (500 mg, 51%); mp: 231.3 °C; ^1^H-NMR: (400.13 MHz, DMSO-*d*_6_) δ = 6.85 (60%, H_4_), 6.90–7.24 (40% H_4_, H_6_, H_7_, H_5′_, H_6′_), 7.24–7.51 (H_2′_, H*m*, H*p*), 7.76 (40%, H*o*), 7.80 (60%, H*o*), 10.03 (40%, OH), 10.12 (60%, OH), 13.05 (60%, NH), 13.25 (40%, NH); ^13^C-NMR: (100.62 MHz, DMSO-*d*_6_) δ = 99.3 (40%, C_4_), 100.0 (60%, C_4_), 113.4 (^2^*J*_F_ = 18.6, C_2′_), 114.0 (60%, C_6_), 117.9 (60%, C_5′_), 119.7 (40%, C_6_ and 40%, C_5′_), 123.1 (40%, C_6′_), 123.4 (60%, C_6′_), 125.1 (C*o*), 127.4 (60%, C*p*), 128.1 (60%, C_7_), 128.5 (40%, C_7_ and 40%, C*p*), 128.6 (60%, C*m*), 128.8 (40%, C*m*), 129.0 (60%, C_1′_ and 40%, C*i*), 129.3 (40%, C_1′_), 133.6 (60%, C*i*), 142.2 (60%, C_5_), 142.8 (40%, C_5_), 144.5 (^2^*J*_F_ = 12.7, 40%, C_4′_), 145.0 (^2^*J*_F_ = 12.3, 60%, C_4′_), 150.9 (60%, C_3_), 151.1 (40%, C_3_), 151.2 (^1^*J*_F_ = 240.8, C_3′_). ^15^N-NMR: (40.54 MHz, DMSO-*d*_6_) δ = −179.7 (40%, N_1_), −177.3 (60%, N_1_). ^19^F-NMR: (376 MHz, DMSO-*d*_6_) δ = −136.0 (^3^*J*_H2__′_ = ^4^*J*_H5__′_ = 10.8, 60%, F_3_), −136.2 (^3^*J*_H2__′_ = ^4^*J*_H5__′_ = 10.2, 40%, F_3_); ^13^C-NMR: (100.73 MHz, CPMAS) δ = 97.9 (C_4_), 109.1 (C_2′_), 114.7 (C_5′_), 122.9 (C_6′_), 125.6 (C_6_), 126.3 (C*o*), 128.1 (C*m*, C*p*), 130.4 (C_7_), 131.6 (C_1′_), 132.3 (C*i*), 143.1 (C_5_), 144.9 (C_4′_), 151.8 (C_3_), 156.9/154.6 (C_3′_); ^15^N-NMR: (40.60 MHz, CPMAS) δ = −120.0 (N_2_), −168.5 (N_1_); ^19^F-NMR: (376.94 MHz, MAS ) δ = −129.2; Anal. calcd. for C_17_H_13_FN_2_O: C 72.85, H 4.67, N 9.99. Found: C 72.54, H 4.70, N 9.89.

*(E)-3(5)-[β-(2,4-Difluoro-3-hydroxyphenyl)-ethenyl]-5(3)-phenyl-1H-pyrazole* (**14**). Compound **14** was prepared from (*E*)-5-(2,4-difluoro-3-hydroxyphenyl)-1-phenylpent-4-ene-1,3-dione. The compound was purified by column chromatography using ethyl acetate/hexane as eluent (7:3) to give a pale yellow solid. Yield: (319 mg, 59%); mp: 195.9 °C; ^1^H-NMR: (400.13 MHz, DMSO-*d*_6_) δ = 6.98–7.23 (H_4_, H_6_, H_7_, H_5′_, H_6′_), 7.30 (H*p*), 7.42 (H*m*), 7.80 (H*o*), 10.30 (OH), 13.17 (54%, NH), 13.33 (46%, NH); ^13^C-NMR: (100.62 MHz, DMSO-*d*_6_) δ = 99.9 (46%, C_4_), 100.7 (54%, C_4_), 111.9 (^2^*J*_F_ = 18.6, C_5′_), 115.9 (^3^*J*_F_ = 8.2, ^3^*J*_F_ = 3.6, C_6′_), 117.8 (C_6_), 120.7 (46%, C_1′_), 121.3 (54%, C_1′_), 123.6 (C_7_) 125.1 (C*o*), 127.6 (C*p*), 128.8 (C*m*), 133.5 (C*i*), 134.0 (^2^*J*_F_ = ^2^*J*_F_ = 16.1, C_3′_), 141.8 (54%, C_5_), 143.0 (46%, C_5_), 150.2 (^1^*J*_F_ = 245.6, ^3^*J*_F_ = 5.8, C_2′_), 151.0 (54%, C_3_), 151.7 (^1^*J*_F_ = 240.6, C_4′_), 153.3 (46%, C_3_); ^15^N-NMR: (40.54 MHz, DMSO-*d*_6_) δ = −178.3 ( 54%, N1); ^19^F-NMR: (376.50 MHz, DMSO-*d*_6_) δ = −137.0 (46%, F_2_), −136.4 (54%, F_2_), −132.8 (46%, F_4_), −132.1 (54%, F_4_); ^13^C-NMR: (100.73 MHz, CPMAS) δ = 97.1 (C_4_), 111.2 (C_5′_), 119.4 (C_6′_), 121.7 (C_6_, C_1′_), 123.6 (C_7_), 126.8 (C*i*, C*o*), 129.0 (C*p*), 132.3 (C*m*), 135.5 (C_3′_), 142.5 (C_5_), 150.1 (C_2′_), 153.1 (C_4′_), 153.7 (C_3_); ^15^N-NMR: (40.60 MHz, CPMAS) δ = −191.7 (N_1_), −93.9 (N_2_); ^19^F-NMR: (376.94 MHz, MAS) δ = −130.7 (F_2_), −126.8 (F_4_). Anal. calcd. for C_17_H_12_F_2_N_2_O: C 68.45, H 4.05, N 9.39. Found: C 68.57, H 4.09, N 9.22.

*(E)-3(5)-[β-(2,5-Difluoro-4-hydroxyphenyl)-ethenyl]-5(3)-phenyl-1H-pyrazole* (**15**). Compound **15** was prepared from (*E*)-5-(2,5-difluoro-4-hydroxyphenyl)-1-phenylpent-4-ene-1,3-dione. The compound was purified by column chromatography using ethyl acetate/hexane as eluent (5:5) to give a pale yellow solid. Yield: (319 mg, 59%); mp: 232.6 °C; ^1^H-NMR: (400.13 MHz, DMSO-*d*_6_) δ = 6.78 (^4^*J*_F5_ = 7.4, ^3^*J*_F2_ = 11.6, H_3′_), 6.91(60% H_4_), 6.99 (40% H_4_), 7.06 (^3^*J*_H7_ = 16.6, H_6_), 7.15 (^3^*J*_H6_ = 16.6, H_7_), 7.31 (H*p*), 7.42 (H*m*), 7.58 (H_6′_), 7.80 (H*o*), 10.60 (OH), 13.12 (60%, NH), 13.28 (40%, NH); ^13^C-NMR: (100.62 MHz, DMSO-*d*_6_) δ = 99.6 (40%, C_4_), 100.6 (60%, C_4_), 105.0 (^2^*J*_F2_ = 26.6, C_3′_), 113.4 (^2^*J*_F5_ = 21.1, ^3^*J*_F2_ = 5.4, C_6′_), 114.9 (60%, C_1′_), 115.3 (40%, C_1′_), 116.2 (60%, C_6_), 120.1 (40% C_6_), 120.8 (60% C_7_), 122.0 (40% C_7_), 125.1 (C*o*), 127.5 (60%, C_p_), 128.3 (40%, C*p*), 128.7 (C*m*), 133.5 (C*i*), 142.0 (60%, C_5_), 143.0 (40%, C_5_), 145.7 (C_4′_), 148.1 (^1^*J*_F_ = 236.9, C_5′_), 151.0 (C_3_), 155.7 (^1^*J*_F_ = 244.6, C_2′_); ^15^N-NMR: (40.54 MHz, DMSO-*d*_6_) δ = −178.6 ( 60%, N1); ^19^F-NMR: (376.50 MHz, DMSO-*d*_6_) δ = −140.6 (F_5_), −122.1(40% F_2_), −121.3 (60%, F_2_); ^13^C-NMR: (100.73 MHz, CPMAS) δ = 98.4 (C_4_), 109.8 (C_3′_), 110.6 (C_6′_), 116.8 (C_6_), 119.8 (C_1′_), 124.9/123.7 (C_7_), 128.1 (C*o*), 128.9 (C*m*), 129.3 (C*i*), 130.9 (C*p*), 143.9 (C_5_), 148.0 (C_4′_), 148.9 (C_3_), 153.5/150.9 (C_5′_), 157.5/154.8 (C_2′_); ^15^N-NMR: (40.60 MHz, CPMAS) δ = −141.1 (N_2_), −152.2 (N_1_); ^19^F-NMR: (376.94 MHz, MAS) δ = −134.8 (F_5_), −114.2 (F_2_). Anal. calcd. for C_17_H_12_F_2_N_2_O: C 68.45, H 4.05, N 9.39. Found: C 68.14, H 4.16, N 9.20.

*(E)-3(5)-[β-(4-Fluoro-3-methoxyphenyl)-ethenyl]-5(3)-phenyl-1H-pyrazole* (**16**). Compound **16** was prepared from (*E*)-5-(4-fluoro-3-methoxyphenyl)-1-phenylpent-4-ene-1,3-dione. The compound was obtained as yellow crystals after recrystallization from CH_2_Cl_2_/hexane. Yield: (499 mg, 55%); mp: 150.3 °C; ^1^H-NMR: (400.13 MHz, DMSO-*d*_6_) δ = 3.90 (OCH_3_), 6.90 (57%, H_4_), ~7.0 (43%, H_4_), 7.06–7.26 (H_6_, H_7_, H_5′_, H_6′_) 7.20–7.50 (H_2′_, H*m*, H*p*), 7.77 (43%, H*o*), 7.80 (57%, H*o*), 13.10 (57%, NH), 13.29 (43%, NH); ^13^C-NMR: (100.62 MHz, DMSO-*d*_6_) δ = 56.0 (OCH_3_), 99.4 (43%, C_4_), 100.3 (57%, C_4_), 110.9 (C_2′_), 115.9 ( ^2^*J*_F_ ≈ 18, 57% C_5′_, 57% C_6_), 116.0 ( ^2^*J*_F_ ≈ 23, 43%, C_5′_), 119.4 (57% C_6′_, 43% C_6_), 121.5 (43%, C_6′_), 125.1 (C*o*), 127.4 (57%, C*p*), 128.1 (43%, C*p*), 128.3 (43%, C_7_), 128.7 (57% C*m*), 128.9 (57%, C_7_), 129.0 (C_1′_, 43% C*m*), 133.5 (57%, C*i*), 134.1 (43%, C*i*), 142.0 (57%, C_5_), 142.9 (43%, C_5_), 147.4 (^2^*J*_F_ = 10.9, C_3′_), 150.9 (C_3_), 151.1 (^1^*J*_F_ ≈ 246.1, 43%, C_4′_), 151.3 (^1^*J*_F_ ≈ 249.5, 57%, C_4′_); ^15^N-NMR: (40.54 MHz, DMSO-*d*_6_) δ = −179.2 (43%, N_1_), −177.6 (57%, N_1_); ^19^F-NMR: (376.50 MHz, DMSO-*d*_6_) δ = −136.3 (43%), −135.4 (57%); ^13^C-NMR: (100.76 MHz, CPMAS) δ = 56.7 (OCH_3_), 102.7 (C_4_), 113.8 (C_2′_), 114.2 (C_5′_), 115.0 (C_6_), 118.4 (C_6′_), 124.3/127.2 (C*o*), 128.3 (C*m*, C*p*), 130.5 (C_7_), 133.6 (C_1′_, C*i*), 142.1 (C_5_), 148.2 (C_3′_), 152.1 (C_3_, C_4′_); ^15^N-NMR: (40.60 MHz, CPMAS) δ = −181.1(N_1_), −94.7 (N_2_); ^19^F-NMR: (376.94 MHz, MAS) δ = −132.6; Anal. calcd. for C_18_H_15_FN_2_O: C 73.45, H 5.14, N 9.52. Found: C 73.13, H 5.13, N 9.60.

### 3.4. In Vitro nNOS, iNOS and eNOS Activities Determination

l-Arginine, l-citrulline, *N*-(2-hydroxymethyl)piperazine-*N*′-(2-ethanesulfonic acid) (HEPES), d-l-dithiothreitol (DTT), leupeptin, aprotinin, pepstatin, phenylmethyl-sulfonyl fluoride (PMSF), hypoxantine-9-β-d-ribofuranosid (inosine), ethylene glycol-bis-(2-aminoethylether)-*N*,*N*,*N*′,*N*′-tetraacetic acid (EGTA), bovine serum albumin (BSA), Dowex-50W (50 X 8–200), FAD, NADPH and 5,6,7,8-tetrahydro-l-biopterin dihydrochloride (H_4_-biopterin) were obtained from Sigma-Aldrich Química (Madrid, Spain). l-[^3^*H*]-arginine monohydrochloride (45–70 Ci/mmol, 1 mCi/mL) was obtained from Perkin Elmer Life Sciences (PerkinElmer). Tris(hydroxymethyl)aminomethane (Tris-HCl) and calcium chloride were obtained from Merck (Darmstadt, Germany). Calmodulin from bovine brain was obtained from Alexis Biochemicals (Enzo Life Sciences, Grupo Taper, Seville, Spain), recombinant iNOS (specific activity of 3.9 μmol/min/mg protein) and eNOS (Cayman Chemical, specific activity 3 μmol/min/mg protein) were obtained from Cayman Chemical (Vitro S.A, Madrid, Spain).

For nNOS activity determination, C57BL/6 mice (3-months old, 25–30 g) (The Jackson Laboratory, Bar Harbor, Maine, ME, USA) were housed in the animal facility of the University of Granada under specific pathogen-free conditions, and in a controlled 12 h light/dark cycle at 22 ± 2 °C. Mice had unlimited access to water and rodent chow until the day of the experiment. All experiments were performed according to the Spanish Government Guide and the European Community Guide for animal care. The experimental paradigm was published elsewhere [81]. Briefly, mice were killed by cervical dislocation and brain was quickly collected, washed, and homogenized in an ice-cold buffer (51 mM Tris, 1 mM DTT, 10 μg/mL leupeptin, 10 μg/mL pepstatin, 10 μg/mL aprotinin, 1 mM PMSF, pH 7.6). The crude homogenate was centrifuged twice at 1000 *g* at 4 °C, and sonicated (10 s × 6). Aliquots of the supernatant were either stored at −80 °C for total protein quantification [82] or immediately used for nNOS activity measurement. For iNOS and eNOS activity determination, recombinant enzymes were used.

The rationale for the use of different sources of pure NOS isoforms comes from their relative abundance in normal tissue. Neuronal NOS (nNOS) is abundant in the brain, and it is a suitable source to obtain a pure, endogenous form, allowing the study of its kinetic properties [83]. However, iNOS is not constitutively expressed in normal tissue (or it is expressed at very low level), and it is only present in high amounts during inflammation; therefore, it is much more convenient to obtain it from a commercial source. In the case of eNOS, it is also expressed at low levels in vascular areas and, although it may be induced during inflammation, this seems to only occur in neutrophils. Thus, endogenous source of eNOS is also not appropriate for *in vitro* assays.

NOS activity was measured following the conversion of l-^3^*H*-arginine to l-^3^*H*-citrulline according to the Bredt *et al.* protocol [71]. Enzyme activity was referred as pmol l-^3^*H*-citrulline/min/mg protein. The final incubation volume was 100 μL and consisted of 10 μL of an aliquot of homogenized brain (for nNOS measurement) or recombinant enzyme (for iNOS or eNOS measurement) added to a buffer with a final concentration of 25 mM Tris-HCl, 0.5 mM DTT, 30 μM H_4_-biopterin, 10 μM FAD, 0.5 mM inosine, 1 mg/mL BSA, 0.5 mM CaCl_2_ (or 1 mM for iNOS measurement), 10 μM l-arginine, and 50 nM l-[^3^*H*]-arginine, 10 μg/mL calmodulin (only for constitutive isoforms), at pH 7.6. Final volume also include 10 μL of each pyrazole compound dissolved in EtOH or DMSO to give a final concentration of 1 mM or 50 μM, respectively. The activity of the different NOS isoforms was also assayed in the presence of each vehicle (EtOH or DMSO) to discard any non-specific effect of the compounds. Neither EtOH nor DMSO had significant effects of the NOS activity measured. The reaction was started by the addition of 10 μL of NADPH (0.75 mM final) and continued for 30 min at 37 °C. Control incubations were performed by the omission of NADPH. The reaction was stopped adding 400 μL of cold 0.1 M HEPES, 0.1 M EGTA, and 0.175 mg/mL l-citrulline, pH 5.5. The reaction mixture was decanted onto a 2 mL column packet with Dowex-50W ion-exchange resin (Na^+^ form) and eluted with 1.2 mL of water. l-^3^*H*-citrulline was quantified by liquid scintillation counting in a Beckman LS-6000 system (Beckman Coulter, Fullerton, CA, USA). The retention of l-[^3^*H*]-arginine in this process was greater than 98%. Specific enzyme activity was determined by subtracting the control value. Statistical analysis: Data are expressed as the mean ± SEM. Statistical analysis was performed by Student’s *t*-test. A *p* < 0.05 was considered statistically significant.

### 3.5. Single-Crystal X-ray Analysis

Data collection for **13** and **16** was carried out at room temperature on a Bruker Smart CCD diffractometer and on a Xcalibur, Atlas CCD diffractometer for **12**, using in all cases graphite-monochromated Mo-Kα radiation (λ = 0.71073 Å) operating at 50 kV and 40 mA for **12**, at 50 kV and 30 mA for **13** and **16**. The exposure times were 3.37 s for **12** and 20 s for **13** and **16** in omega.

A summary of the fundamental crystal and refinement data is given in Table 6. The structures were solved by direct methods and refined by full-matrix least-squares procedures on F^2^ (SHELXL-97) [84]. All non-hydrogen atoms were refined anisotropically. All hydrogen atoms were included in their calculated positions and refined riding on the respective carbon atoms.

**Table 6:** Crystal data and Refinement Data for **12**, **13** and **16**.

Crystal Data	12	13	16
CCDC	1040404	1040405	1040406
Empirical formula	C_17_H_13_FN_2_O	C_17_H_13_FN_2_O	C_18_H_15_FN_2_O
Formula wt	280.29	280.29	294.32
Crystal system	Monoclinic	Monoclinic	Monoclinic
Space group	*P*2_1_/*n*	*P*2_1_/*n*	*P*2_1_/*c*
a/Å	12.5535(10)	12.3701(12)	16.245(5)
b/Å	7.4573(4)	7.4538(7)	12.187(4)
c/Å	15.0727(11)	15.2521(15)	7.559(2)
a α/°	90	90	90
b β/°	106.759(8)	105.101(2)	94.205(6)
γ/°	90	90	90
*V*/Å^3^	1351.1(2)	1357.7(2)	3280.0(3)
*Z*	4	4	4
D_c_/g/cm^3^	1.378	1.371	1.310
μ(Mo-Ka)/mm^−1^	0.097	0.096	0.091
F(000)	584	584	616
θ range/°	3.39 to 25.01	1.90 to 27.00	1.26 to 27.00
Index ranges	−14,−8,−14 to 14, 8, 17	−15,−9,−19 to 15, 8, 17	−20,−15,−9 to 20, 15, 5
Reflections collected	5657	10842	13044
Unique reflections	2370	2972	3259
[Rint]	[0.0278]	[0.0564]	[0.0593]
Completeness to θ	99.7%	99.7%	99.9%
Data/restraints/params	2370/0/190	2972/0/192	3259/0/199
Goodness-of-fit on F^2^	0.997	0.997	0.995
R1	0.0437	0.0507	0.0379
(reflns obsd) [I > 2 *s*(I)] ^[a]^	(1664)	(1415)	(1548)
*w*R2 (all data) ^[b]^	0.1218	0.1631	0.0867

^[a]^ R1 = Σ||F_o_| − |F_c_||/Σ|F_o_|; ^[b]^ *w*R2 = {Σ[*w*(F_o_^2^ − F_c_^2^)^2^]/Σ[*w*(F_o_^2^)^2^]}.

CCDC 1040404, 1040405 and 1040406 contain the supplementary crystallographic data for this paper. These data can be obtained free of charge via http://www.ccdc.cam.ac.uk/conts/retrieving.html (or from the CCDC, 12 Union Road, Cambridge CB2 1EZ, UK; Fax: +44 1223 336033; E-mail: deposit@ccdc.cam.ac.uk).

## 4. Conclusions

We have evaluated and compared the potency and selectivity of two series of curcuminoid pyrazoles, compounds **7**–**11** without fluorine and compounds **12**–**16** containing fluorine, as inhibitors of the different nitric oxide synthase (NOS) isoforms, and found that as a whole they are mostly iNOS inhibitors, contrary to the one derived from curcumin, pyrazole **1**, which is predominantly eNOS. One of the most interesting compounds is (*E*)-3(5)-[β-(3-fluoro-4-hydroxyphenyl)-ethenyl]-5(3)-phenyl-1*H*-pyrazole (**13**), as it is not only the best inhibitor of iNOS, but also more selective towards the other two isoforms.

Tautomerism studies on both series have been achieved by means of multinuclear NMR (DMSO and HMPA) to establish the predominant tautomer, 5-styryl **a** or 3-styryl **b**, in each case. The effect of the 4-methyl group in the pyrazole ring, which increases the average potency in the 3(5)-methyl series and decreases it in the 3(5)-phenyl series, seems related to the fact that, in the first case, there is a change of tautomerism (**7b** *vs.* **8a**), while in the second one, both tautomers are the same (**10a** and **11a**). The most potent eNOS inhibitor, **7**, has the lowest percentage of **a** tautomer.

The analysis of substituent effects on the percentages of NOS inhibition demonstrates the importance of the introduction of fluorine atoms and opens a new avenue to search for new active substances.

## Supplementary Files

Tables containing the complete ^1^H, ^13^C, ^15^N and ^19^F-NMR set of data (chemical shifts and coupling constants) in solution and in solid state. The ORTEP plots (40% ellipsoid probability) of **13** and **16** showing the labeling of their asymmetric units. Figures corresponding to the *in vitro* nNOS, iNOS and eNOS activities determination. The Free–Wilson matrix used to obtain the statistical results of Table 5, can be accessed at: http://www.mdpi.com/1420-3049/20/09/15643/s1.

## References

[1] Claramunt R.M., López C., Pérez-Medina C., Pérez-Torralba M., Elguero J., Escames G., Acuña-Castroviejo D. (2009). Fluorinated indazoles as novel selective inhibitors of nitric oxide synthase (NOS): Synthesis and biological evaluation. Bioorg. Med. Chem..

[2] Elguero J., Alkorta I., Claramunt R.M., López C., Sanz D., Santa María D. (2009). Theoretical calculations of a model of NOS indazole inhibitors: Interaction of aromatic compounds with Zn-porphyrins. Bioorg. Med. Chem..

[3] Claramunt R.M., López C., López A., Pérez-Medina C., Pérez-Torralba M., Alkorta I., Elguero J., Escames G., Acuña-Castroviejo D. (2011). Synthesis and biological evaluation of indazole derivatives. Eur. J. Med. Chem..

[4] Cornago P., Claramunt R.M., Bouissane L., Alkorta I., Elguero J. (2008). A study of the tautomerism of β-dicarbonyl compounds with special emphasis on curcuminoids. Tetrahedron.

[5] Cornago P., Cabildo P., Claramunt R.M., Pinilla E., Torres M.R., Elguero J. (2009). The annular tautomerism of the curcuminoid NH-pyrazoles. New J. Chem..

[6] Claramunt R.M., Bouissane L., Cabildo P., Cornago P., Elguero J., Radziwon A., Medina C. (2009). Synthesis and biological evaluation of curcuminoid pyrazoles as new therapeutic agents in inflammatory bowel disease: Effect on matrix metalloproteinases. Bioorg. Med. Chem..

[7] Cornago P., Cabildo P., Sanz D., Claramunt R.M., Torralba M.C., Torres M.R., Elguero J. (2013). Structures of hemi-curcuminoids in the solid-state and in solution. Eur. J. Org. Chem..

[8] Bredt D.S., Snyder S.H. (1994). Nitric oxide: A physiologic messenger molecule. Annu. Rev. Biochem..

[9] Escames G., León J., Macías M., Khaldy H., Acuña-Castroviejo D. (2003). Melatonin counteracts lipopolysaccharide-induced expression and activity of mitochondrial nitric oxide synthase in rats. FASEB J..

[10] Lacza Z., Pankotai E., Csordás A., Kiss L., Horváth E.M., Kollai M., Busija D.W., Szabó C. (2006). Mitochondrial NO and reactive nitrogen species production: Does mtNOS exist?. Nitric Oxide.

[11] Laskin J.D., Laskin D.L. (1999). Cellular and Molecular Biology of Nitric Oxide.

[12] Suaifan G.A., Goodyer C.L.M., Threadgill M.D. (2010). Synthesis of *N*-(methoxycarbonylthienylmethyl) thioureas and evaluation of their interaction with inducible and neuronal nitric oxide synthase. Molecules.

[13] Handy R.L.C., Wallace P., Gaffen Z.A., Whitehead K.J., Moore P.K. (1995). The antinociceptive effect of 1-(2-trifluoromethylphenyl) imidazole (TRIM), a potent inhibitor of neuronal nitric oxide synthase *in vitro*, in the mouse. Br. J. Pharmacol..

[14] Doucet M.V., Levine H., Dev K.K., Harkin A. (2013). Small-molecule inhibitors at the PSD-95/nNOS interface have antidepressant-like properties in mice. Neuropsychopharmacology.

[15] Shankaran K., Donnelly K.L., Shah S.K., Humes J.L., Pacholok S.G., Grant S.K., Green B.G., MacCoss M. (1997). Inhibition of nitric oxide synthase by benzoxazolones. Bioorg. Med. Chem. Lett..

[16] Raman C.S., Martásek H., Li P., Babu B.R., Griffith O.W., Masters B.S., Poulos T.L. (2001). Implications for isoform-selective inhibitor design derived from the binding mode of bulky isothioureas to the heme domain of endothelial nitric-oxide synthase. J. Biol. Chem..

[17] Moore P.K., Wallace P., Gaffen Z., Hart S.L. (1993). Characterization of the novel nitric oxide synthase inhibitor 7-nitro indazole and related indazoles: Antinociceptive and cardiovascular effects. Br. J. Pharmacol..

[18] Babbedge R.C., Bland-Ward P.A., Hart S.L., Moore P.K. (1993). Inhibition of rat cerebellar nitric oxide synthase by 7-nitro indazole and related substituted indazoles. Br. J. Pharmacol..

[19] Cottyn B., Acher F., Ramassamy B., Alvey L., Lepoivre M., Frapart Y., Stuehr D., Mansuy D., Boucher J.L., Vichard D. (2008). Inhibitory effects of a series of 7-substituted-indazoles toward nitric oxide synthases: Particular potency of 1*H*-indazole-7-carbonitrile. Bioorg. Med. Chem..

[20] Carrión M.D., Chayah M., Entrena A., López A., Gallo M.A., Acuña-Castroviejo D., Camacho M.E. (2013). Synthesis and biological evaluation of 4,5-dihydro-1*H*-pyrazole derivatives as potential nNOS/iNOS selective inhibitors. Part 2: Influence of diverse substituents in both the phenyl moiety and the acyl group. Bioorg. Med. Chem..

[21] Ji H., Stanton B.Z., Igarashi J., Li H., Martásek P., Roman L.J., Poulos T.L., Silverman R.B. (2008). Minimal pharmacophoric elements and fragment hoping, an approach directed at molecular diversity and isozyme selectivity. Design of selective neuronal nitric oxide synthase inhibitors. J. Am. Chem. Soc..

[22] Ji H., Delker S.L., Li H., Martásek P., Roman L.J., Poulos T.L., Silverman R.B. (2010). Exploration of the active site of neuronal nitric oxide synthase by the design and synthesis of pyrrolidinomethyl 2-aminopyridine derivatives. J. Med. Chem..

[23] Cinelli M.A., Li H., Chreifi G., Martásek P., Roman L.J., Poulos T.L., Silverman R.B. (2014). Simplified 2-aminoquinoline-based scaffold for potent and selective neuronal nitric oxide synthase inhibition. J. Med. Chem..

[24] Kang S., Tang W., Li H., Chreifi G., Martásek P., Roman L.J., Poulos T.L., Silverman R.B. (2014). Nitric oxide synthase inhibitors that interact with both heme propionate and tetrahydrobiopterin show high isoform selectivity. J. Med. Chem..

[25] Li H., Jamal J., Delker S., Plaza C., Ji H., Jing Q., Huang H., Kang S., Silverman R.B., Poulos T.L. (2014). The mobility of a conserved tyrosine residue controls isoform-dependent enzyme-inhibitor interactions in nitric oxide synthases. Biochemistry.

[26] Mukherjee P., Li H., Sevrioukova I.F., Chreifi G., Martásek P., Roman L.J., Poulos T.L., Silverman R.B. (2015). Novel 2,4-disubstituted pyrimidines as potent, selective, and cell-permeable inhibitors of neuronal nitric oxide synthase. J. Med. Chem..

[27] Aggarwal B.B., Kumar A., Aggarwal M.S., Shihodia S., Bagchi D., Preuss H.G. (2004). Curcumin Derived from Turmeric (*Curcuma longa*): A Spice for All Seasons. Phytopharmaceuticals in Cancer Chemoprevention.

[28] Maheshwari R.K., Singh A.K., Gaddipati J., Srimal R.C. (2006). Multiple biological activities of curcumin: A short review. Life Sci..

[29] Zhou H., Beevers C.S., Huang S. (2011). The targets of curcumin. Curr. Drug Targets.

[30] Esatbeyoglu T., Huebbe P., Ernst I.M.A., Chin D., Wagner A.E., Rimbach G. (2012). Curcumin—From molecule to biological function. Angew. Chem. Int. Ed..

[31] Wolf L.K. (2012). Turmeric-derived compound curcumin may treat Alzheimer’s. Chem. Eng. News.

[32] Prasad S., Gupta S.C., Tyagi A.K., Aggarwal B.B. (2014). Curcumin, a compound of golden spice: From bedside to bench and back. Adv. Biotech.

[33] Tizabi Y., Hurley L.L., Qualls Z., Akinfiresoye L. (2014). Relevance of the anti-inflammatory properties of curcumin in neurodegenerative diseases and depression. Molecules.

[34] Liu K., Zhang D., Chojnacki J., Du Y., Fu H., Grant S., Zhang S. (2013). Design and biological characterization of hybrid compounds of curcumin and thalidomide for multiple myeloma. Org. Biomol. Chem..

[35] Chojnacki J.E., Liu K., Yan X., Toldo S., Selden T., Estrada M., Rodríguez-Franco M.I., Halquist M.S., Ye D., Zhang S. (2014). Discovery of 5-(4-hydroxyphenyl)-3-oxo-pentanoic acid [2-(5-methoxy-1H-indol-3-yl)-ethyl]-amide as a neuroprotectant for Alzheimer’s disease by hybridization of curcumin and melatonin. ACS Chem. Neurosci..

[36] Yang C., Wang Z., Ou C., Chen M., Wang L., Yang Z. (2014). A supramolecular hydrogelator of curcumin. Chem. Commun..

[37] Mareeswaran P.M., Babu E., Sathish V., Kim B., Woo S.I., Rajagopal S. (2014). *p*-Sulfonatocalix[4]arene as a carrier for curcumin. New J. Chem..

[38] Mishra M.K., Sanphui P., Ramamurty U., Desiraju G.R. (2014). Solubility-hardness correlation in molecular crystals: curcumin and sulfathiazole polymorphs. Cryst. Growth Des..

[39] Jirásek P., Amslinger S., Heilmann J. (2014). Synthesis of natural and non-natural curcuminoids and their neuroprotective activity against glutamate-induced oxidative stress in HT-22 Cells. J. Nat. Prod..

[40] Flynn D.L., Belliotti T.R., Boctor A.M., Connor D.T., Kostlan C.R., Nies D.E., Ortwine D.F., Schrier D.J., Sircar J.C. (1991). Styrylpyrazoles, styrylisoxazoles, and styrylisothiazoles. Novel 5-lipoxygenase and cyclooxygenase inhibitors. J. Med. Chem..

[41] Shim J.S., Kim D.H., Jung H.J., Kim J.H., Lim D., Lee S.K., Kim K.W., Ahn J.W., Yoo J.S., Rho J.R. (2002). Hydrazinocurcumin, a novel synthetic curcumin derivative, is a potent inhibitor of endothelial cell proliferation. Bioorg. Med. Chem..

[42] Ishida J., Ohtsu H., Tachibana Y., Nakanishi Y., Bastow K.F., Nagai M., Wang H.K., Itokawa H., Lee K.H. (2002). Antitumor agents. Part 214: Synthesis and evaluation of curcumin analogues as cytotoxic agents. Bioorg. Med. Chem..

[43] Caldarelli A., Penucchini E., Caprioglio D., Genazzani A.A., Minassi A. (2013). Synthesis and tubulin-binding properties of non-symmetrical click C5-curcuminoids. Bioorg. Med. Chem..

[44] Selvam C., Jachak S.M., Thilagavathia R., Chakraborti A.K. (2005). Design, synthesis, biological evaluation and molecular docking of curcumin analogues as antioxidant, cyclooxygenase inhibitory and anti-inflammatory agents. Bioorg. Med. Chem. Lett..

[45] Liu Y., Dargush R., Maher P., Schubert D. (2008). A broadly neuroprotective derivative of curcumin. J. Neurochem..

[46] Maher P., Akaishi T., Schubert D., Abe K. (2010). A pyrazole derivative of curcumin enhances memory. Neurobiol. Aging.

[47] Lapchak P.A., McKim J.M. (2011). CeeTox™ analysis of CNB-001 a novel curcumin-based neurotrophic/neuroprotective lead compound to treat stroke: comparison with NXY-059 and Radicut. Transl. Stroke Res..

[48] Chen Q., Prior M., Dargush R., Roberts A., Riek R., Eichmann C., Chiruta C., Akaishi T., Abe K., Maher P. (2011). A novel neurotrophic drug for cognitive enhancement and Alzheimer’s disease. PLoS ONE.

[49] Narlawar R., Pickhardt M., Leuchtenberger S., Baumann K., Krause S., Dyrks T., Weggen S., Mandelkow E., Schmidt B. (2008). Curcumin-derived pyrazoles and isoxazoles: Swiss army knives or blunt tools for Alzheimer’s disease?. ChemMedChem.

[50] Lee D.W., Park J.H., Yoon S.S. (2014). Synthesis and biological evaluation of curcumin analogs as antiplatelet inhibitor. Bull. Korean Chem. Soc..

[51] Brouet I., Ohshima H. (1995). Curcumin, an anti-tumor promoter and anti-inflammatory agent, inhibits induction of nitric oxide synthase in activated macrophages. Biochem. Biophys. Res. Commun..

[52] Chan M.M.Y., Huang H.I., Fenton M.R., Fong D. (1998). *In vivo* inhibition of nitric oxide synthase gene expression by curcumin, a cancer preventive natural product with anti-inflammatory properties. Biochem. Pharmacol..

[53] Onoda M., Inano H. (2000). Effect of curcumin on the production of nitric oxide by cultured rat mammary gland. Nitric Oxide.

[54] Chen Y.C., Shen S.C., Lee W.R., Hou W.C., Yang L.L., Lee T.J.F. (2001). Inhibition of NOS inhibitors and lipopolysaccharide induced inducible nitric oxide synthase and cyclooxygenase 2 gene expressions by rutin, quercetin, and quercetin pentaacetate in RAW264.7 macrophages. J. Cell. Biochem..

[55] Surh Y.J., Chun K.S., Cha H.H., Han S.S., Keum Y.S., Park K.K., Lee S.S. (2001). Molecular mechanisms underlying chemopreventive activities of anti-inflammatory phytochemicals: Down-regulation of COX-2 and iNOS through suppression of NF-kappa B activation. Mutat. Res..

[56] Inano H., Onoda M. (2003). Role of nitric oxide in radiation-induced initiation of mammary tumorigenesis in rats. Nitric Oxide.

[57] Chen J., Tang X.Q., Zhi J.L., Cui Y., Yu H.M., Tang E.H., Sun S.N., Feng J.Q., Chen P.X. (2006). Curcumin protects PC12 cells against 1-methyl-4-phenylpyridinium ion-induced apoptosis by bcl-2-mitochondria-ROS-iNOS pathway. Apoptosis.

[58] Kuhad A., Pilkhwal S., Sharma S., Tirkey N., Chopra K. (2007). Effect of curcumin on inflammation and oxidative stress in cisplatin-induced experimental nephrotoxicity. J. Agric. Food Chem..

[59] Menon V.P., Sudheer A.R., Aggarwal B.B., Surh Y.J., Shishodia S. (2007). The Molecular Targets and Therapeutic Uses of Curcumin in Health and Disease.

[60] Ben P., Liu J., Lu C., Xu Y., Fu J., Huang H., Zhang Z., Gao Y., Luo L., Yin Z. (2011). Curcumin promotes degradation of inducible nitric oxide synthase and suppresses its enzyme activity in RAW 264.7 cells. Int. Immunopharmacol..

[61] Pan M.H., Lin-Shiau S.Y., Lin J.K. (2000). Comparative studies on the suppression of nitric oxide synthase by curcumin and its hydrogenated metabolites through down-regulation of IkappaB kinase and NFkappaB activation in macrophages. Biochem. Pharmacol..

[62] Motterlini R., Foresti R., Bassi R., Green C.J. (2000). Curcumin, an antioxidant and anti-inflammatory agent, induces heme oxygenase-1 and protects endothelial cells against oxidative stress. Free Radic. Biol. Med..

[63] Braidy N., Grant R., Adams S., Guillemin G. (2010). Neuroprotective effects of naturally occurring polyphenols on quinolinic acid-induced excitotoxicity in human neurons. FEBS J..

[64] Yu S.Y., Zhang M., Luo J., Zhang L., Shao Y., Li G. (2013). Curcumin ameliorates memory deficits via neuronal nitric oxide synthase in aged mice. Prog. Neuropsychopharmacol. Biol. Psychiatry.

[65] Yamazaki T., Taguchi T., Ojima I., Ojima I. (2009). Unique Properties of Fluorine and Their Relevance to Medicinal Chemistry and Chemical Biology. Fluorine in Medicinal Chemistry and Biology.

[66] Elguero J., Katritzky A.R., Rees C.W., Scriven E.F. (1996). Comprehensive Heterocyclic Chemistry II.

[67] Stanovnik B., Svete J., Neier R. (2002). Science of Synthesis.

[68] Fustero S., Simón-Fuentes A., Sanz-Cervera J.F. (2009). Recent advances in the synthesis of pyrazoles. A review. Org. Prep. Proced. Int..

[69] Claramunt R.M., Cornago P., Torres V., Pinilla E., Torres M.R., Samat A., Lokshin V., Valés M., Elguero J. (2006). The structure of pyrazoles in the solid state: A combined CPMAS, NMR, and crystallographic study. J. Org. Chem..

[70] Oki M. (1985). Applications of Dynamic NMR Spectroscopy to Organic Chemistry.

[71] Bredt D.S., Snyder S.H. (1989). Nitric oxide mediates glutamate-linked enhancement of cGMP levels in the cerebellum. Proc. Natl. Acad. Sci. USA.

[72] Hansch C., Leo A. (1995). Exploring QSAR.

[73] Free S.M., Wilson J.W. (1964). A mathematical contribution to structure-activity studies. J. Med. Chem..

[74] Fujita T., Ban T. (1971). Structure–activity study of phenethylamines as substrates of biosynthetic enzymes of sympathetic transmitters. J. Med. Chem..

[75] Alkorta I., Rozas I., Elguero J. (2001). Molecular complexes between silicon derivatives and electron-rich groups. J. Phys. Chem..

[76] Iglesias-Sánchez J.C., Santa María D., Claramunt R.M., Elguero J. (2010). Molecular recognition studies on naphthyridine derivatives. Molecules.

[77] Berger S., Braun S. (2004). 200 and More NMR Experiments: A Practical Course.

[78] Murphy P.D. (1983). Improvement in the cross-polarization NMR experiment for suppression of rigid protonated carbons. J. Magn. Reson..

[79] Murphy P.D. (1985). Pulse sequences for the selective observations of nonprotonated and methyl carbon NMR resonances in solids. J. Magn. Reson..

[80] Alemany L.B., Grant D.M., Alger T.D., Pugmire R.J. (1983). Cross polarization and magic angle sample spinning NMR spectra of model organic compounds. 3. Effect of the ^13^C-^1^H dipolar interaction on cross polarization and carbon-proton dephasing. J. Am. Chem. Soc..

[81] Crespo E., Macías M., Pozo D., Escames G., Martin M., Vives F., Guerrero J.M., Acuña-Castroviejo D. (1999). Melatonin inhibits expression of the inducible NO synthase II in liver and lung and prevents endotoxemia in lipopolysaccharide-induced multiple organ dysfunction in rats. FASEB J..

[82] Lowry O.H., Rosebrough N.J., Farr A.L., Randall R.J. (1951). Protein measurement with the Folin phenol reagent. J. Biol. Chem..

[83] León J., Macías M., Escames G., Camacho E., Khaldy H., Martín M., Espinosa A., Gallo M.A., Acuña-Castroviejo D. (2000). Structure-related inhibition of calmodulin-dependent neuronal nitric-oxide synthase activity by melatonin and synthetic kynurenines. Mol. Pharmacol..

[84] (1997). SHELX, 97.

