# Supplementary material for: Fluorination Effects on NOS Inhibitory Activity of Pyrazoles Related to Curcumin
Source: Molecules. 2015 Aug 28;20(9):15643–65. doi: 10.3390/molecules200915643 (PMC6332466; doi:10.3390/molecules200915643)
Supplement: Supplementary file 1 [file molecules-20-15643-s001.pdf]

Supplementary Material

NMR Parameters

Solution NMR spectra were recorded on a Bruker DRX 400 (9.4 Tesla, 400.13 MHz for ^{1}H, 100.62 MHz for ^{13}C, 40.54 MHz for ^{15}N and 376.50 MHz for ^{19}F) spectrometer with a 5-mm inverse-detection H-X probe equipped with a z-gradient coil (^{1}H, ^{13}C, ^{15}N) and with a QNP 5 mm probe (^{19}F), at 295 K. Chemical shifts (δ in ppm) are given from internal solvent, DMSO-d_6 2.49 for ^{1}H and 39.5 for ^{13}C, $CDCl_3$ 7.26 for ^{1}H and 77.0 for ^{13}C, HMPA-d_{18} 2.51 to the upfield multiplet for ^{1}H and 35.8 for ^{13}C. External references were used for ^{15}N and ^{19}F, nitromethane and $CFCl_3$, respectively. Coupling constants (J in Hz) are accurate to ±0.2 Hz for ^{1}H, ±0.8 Hz for ^{19}F and ±0.6 Hz for ^{13}C. Typical parameters for ^{1}H-NMR spectra were spectral width 6500 Hz and pulse width 7.5 μs at an attenuation level of 0 dB. Typical parameters for ^{19}F-NMR spectra were spectral width 55 kHz, pulse width 13.75 μs at an attenuation level of −6 dB and relaxation delay 1 s. Typical parameters for ^{13}C-NMR spectra were spectral width 21 kHz, pulse width 10.6 μs at an attenuation level of −6 dB and relaxation delay 2 s; WALTZ-16 was used for broadband proton decoupling; the FIDs were multiplied by an exponential weighting (lb = 2 Hz) before Fourier transformation. 2D (^{1}H-^{13}C) gs-HMQC and, (^{1}H-^{13}C) gs-HMBC were acquired and processed using standard Bruker NMR software and in non-phase-sensitive mode [77]. Gradient selection was achieved through a 5% sine truncated shaped pulse gradient of 1 ms. Selected parameters for (^{1}H-^{13}C) gs-HMQC and gs-HMBC spectra were spectral width 3500 Hz for ^{1}H and 20.5 kHz for ^{13}C, 1024 × 256 data set, number of scans 2 (gs-HMQC) or 4 (gs-HMBC) and relaxation delay 1 s. The FIDs were processed using zero filling in the F_1 domain and a sine-bell window function in both dimensions was applied prior to Fourier transformation. In the gs-HMQC experiments, GARP modulation of ^{13}C was used for decoupling. Selected parameters for (^{1}H-^{15}N) gs-HMQC spectra were spectral width 6500 Hz for ^{1}H and 12.5 kHz for ^{15}N, 1024 × 256 data set, number of scans 4, relaxation delay 1 s. The FIDs were processed using zero filling in the F_1 domain and a sine-bell window function in both dimensions was applied prior to Fourier transformation. Variable-temperature experiments (DNMR) were recorded with the same spectrometer and a Bruker BVT3000 temperature unit was used to control the temperature of the cooling gas stream and an exchanger to achieve low temperatures.

Solid-state NMR ^{13}C (100.73 MHz) and ^{15}N (40.60 MHz) CPMAS spectra have been obtained on a Bruker WB 400 spectrometer at 300 K using a 4 mm DVT probehead. Samples were carefully packed in a 4 mm diameter cylindrical zirconia rotor with Kel-F end-caps. Operating conditions involved 3.2 μs 90° ^{1}H pulses and decoupling field strength of 86.2 kHz by TPPM sequence. ^{13}C spectra were originally referenced to a glycine sample and then the chemical shifts were recalculated to the Me_4Si (for the carbonyl atom δ (glycine) = 176.1 ppm) and ^{15}N spectra to $^{15}NH_4Cl$ and then converted to nitromethane scale using the relationship: δ ^{15}N (nitromethane) = δ ^{15}N (ammonium chloride) − 338.1 ppm. Typical acquisition parameters for ^{13}C CPMAS were: spectral width, 40 kHz; recycle delay, 30–100 s; acquisition time, 30 ms; contact time, 5 ms; and spin rate, 12 kHz. In order to distinguish protonated and unprotonated carbon atoms, the NQS (Non-Quaternary Suppression) experiment by conventional cross-polarization was recorded; before the acquisition the decoupler is switched off for a very short time of 25 μs [78–80]. And for ^{15}N CPMAS were: spectral width, 40 kHz; recycle delay, 30–100 s; acquisition time, 35 ms; contact time, 6 ms; and spin rate, 6 kHz.

Solid-state ^{19}F (376.94 MHz) NMR spectra have been obtained on a Bruker WB 400 spectrometer using a MAS DVT BL2.5 X/F/H double resonance probehead. Samples were carefully packed in 2.5 mm

diameter cylindrical zirconia rotors with Kel-F end-caps. Samples were spun at the magic angle at rates of 25 kHz and the experiments were carried out at ambient probe temperature. Typical parameters for single pulse ^{19}F MAS NMR spectra were: spectral width, 75 KHz; pulse width, 2.5 μs; recycle delay, 10 s; scans, 128; and spin rate, 25 kHz. The typical acquisition parameters $^{19}F\{^{1}H\}$ MAS were: spectral width, 75 kHz; recycle delay, 10 s; pulse width, 2.5 μs and proton decoupling field strength of 100 kHz by SPINAL-64 sequence; recycle delay, 10 s; acquisition time, 25 ms; 128 scans; and spin rate, 25 kHz. The ^{19}F spectra were referenced to ammonium trifluoroacetate sample and then the chemical shifts were recalculated to the $CFCl_3$ (δ $CF_3COO^{-}NH_4^{+}$= −72.0 ppm).

Table S1. ^{1}H-NMR in solution of pyrazole derivatives (chemical shifts δ in ppm, *J* coupling constants in Hz).

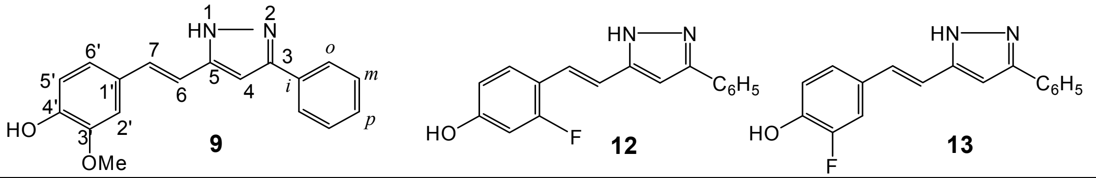

Nuclei	9 [5,6]	12	13			
Solvent (Temp)	DMSO-d_6 300K	DMSO-d_6 295K	DMSO-d_6 300K	DMSO-d_6 290K	DMSO-d_6 360K	HMPA-d_{18} 295K
NH	13.18 (36%)<br>12.96 (64%)	13.23 (33%)<br>13.06 (67%)	13.25 (43%)<br>13.06 (57%)	13.25 (40%)<br>13.05 (60%)	12.75	14.03 (33%)<br>13.80 (67%)
H4	6.88	6.91	6.85	6.85 (major)<br>6.90–7.24	6.80	6.97 (major)<br>7.08–7.19
H6	6.95<br>$^{3}J_{H7}$ = 16.5	6.99<br>$^{3}J_{H7}$ = 16.9	7.89–6.85	6.90–7.24	6.93<br>$^{3}J_{H7}$ = 16.6	7.08–7.19
H7	7.10<br>$^{3}J_{H6}$ = 16.5	7.17<br>$^{3}J_{H6}$ = 16.9	7.89–6.85	6.90–7.24	7.08<br>$^{3}J_{H6}$ = 16.6	7.02–7.04
H2′	7.15	---	7.89–6.85	7.24–7.51	7.30<br>$^{3}J_{F}$ = 12.6<br>$^{4}J_{H6'}$ = 2.1	7.22–7.25
H3′	3.84<br>(OMe)	6.60<br>$^{3}J_{F}$ = 12.8<br>$^{4}J_{H5'}$ = 2.4	---	---	---	---
OH	9.21 (major)<br>9.10 (minor)	10.12	10.13 (major)<br>9.03 (minor)	10.12 (major)<br>10.03 (minor)	9.53	11.15 (major)<br>11.04 (minor)
H5′	6.78<br>$^{3}J_{H6'}$ = 8.1	6.65<br>$^{3}J_{H6'}$ = 8.5<br>$^{4}J_{H3'}$ = 2.4	7.89–6.85	6.90–7.24	6.96<br>$^{3}J_{H6}$ = 8.3<br>$^{4}J_{F}$ = 9.2	7.02–7.04
H6′	6.96<br>$^{3}J_{H5'}$ = 8.1<br>$^{4}J_{H2'}$ = 1.8	7.53	7.89–6.85	6.90–7.24	7.16<br>$^{3}J_{H5}$ = 8.2<br>$^{4}J_{H2'}$ = 2.2	7.08–7.19
H*o*	7.80	7.80	7.79	7.80 (major)<br>7.76 (minor)	7.78	8.02 (minor)<br>7.86 (major)
H*m*	7.43	7.41	7.89–6.85	7.24–7.51	7.41	7.36
H*p*	7.31<br>$^{3}J_{Hm}$ = $^{3}J_{Hm}$ = 6.7	7.30	7.89–6.85	7.24–7.51	7.30	7.22–7.25

Table S1. *Cont.*

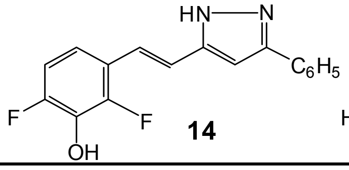

14

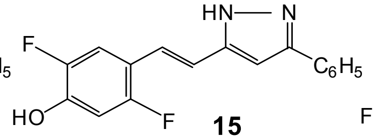

15

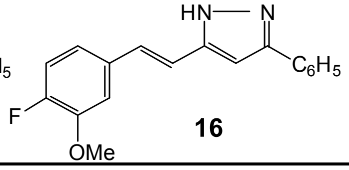

16

Nuclei	14	15	16			
Solvent (Temp)	DMSO-d_6 295K	DMSO-d_6 295K	$CDCl_3$ 300K	DMSO-d_6 295K	DMSO-d_6 360K	HMPA-d_{18} 295K
NH	13.33 (46%)<br>13.17 (54%)	13.28 (40%)<br>13.12 (60%)	n. o.	13.29 (43%)<br>13.10 (57%)	12.84	14.06 (35%)<br>13.94 (65%)
H4	6.98–7.23	6.99 (minor)<br>6.91 (major)	6.75	7.0 (minor)<br>6.90 (major)	6.84	6.92 (major)<br>7.62–6.98 (minor)
H6	6.98–7.23	7.06<br>$^3J_{H7}$ = 16.6	6.97<br>$^3J_{H7}$ = 16.6	7.26–7.06	7.07<br>$^3J_{H7}$ = 16.3	7.62–6.98
H7	6.98–7.23	7.15<br>$^3J_{H6}$ = 16.6	7.05<br>$^3J_{H6}$ = 16.6	7.26–7.06	7.16<br>$^3J_{H6}$ = 16.3	7.62–6.98
H2′	---	---	7.09<br>^4J_F = 8.2<br>$^4J_{H6'}$ = 2.0	7.50–7.20	7.32<br>^4J_F ~ 8.1<br>$^4J_{H6'}$ = 2	7.62–6.98
H3′	---	6.78 (dd)<br>$^4J_{F5'}$ = 7.4<br>$^3J_{F2}$ = 11.6	3.93<br>(OMe)	3.90<br>(OMe)	3.92<br>(OMe)	3.95<br>(OMe)
OH	10.30	10.6 (s, br)	---	---	---	---
H5′	6.98–7.23	---	7.05<br>$^3J_{H6'}$ = 8.3<br>^3J_F = 10.9	7.26–7.06	7.15<br>$^3J_{H6'}$ = 8.5<br>^3J_F = 11.5	7.62–6.98
H6′	6.98–7.23	7.58 (m)	6.99<br>$^3J_{H5'}$ = 8.3<br>^4J_F = 4.7<br>$^4J_{H2'}$ = 2.0	7.26–7.06	7.10<br>^4J_F = 4.7<br>$^3J_{H5'}$ = 8.5<br>$^4J_{H2'}$ = 2	7.62–6.98
H*o*	7.80	7.80 (m)	7.71	7.80 (major)<br>7.77 (minor)	7.78	8.00 (minor)<br>7.84 (major)
H*m*	7.42	7.42 (m)	7.43	7.50–7.20	7.42	7.62–6.98
H*p*	7.30	7.31 (m)	7.36	7.50–7.20	7.31	7.62–6.98

Table S2. ^{13}C- and ^{15}N-NMR in solution and solid state of pyrazole derivatives (chemical shifts δ in ppm, *J* coupling constants in Hz).

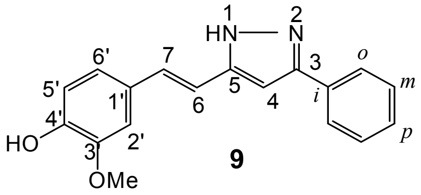

9

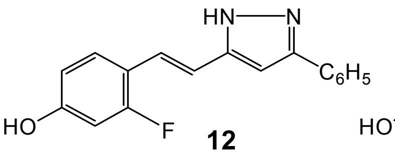

12

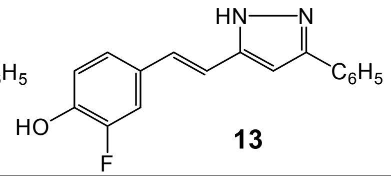

13

**Nuclei**	**9**		**12**		**13**		
**Solvent (Temp)**	**DMSO-d_6 300 K [6]**	**CPMAS 300 K [6]**	**DMSO-d_6 295 K**	**CPMAS 300 K**	**DMSO-d_6 295 K**	**HMPA-d_{18} 295 K**	**CPMAS 300 K**
C3	151.0 (major)<br>151.4 (minor)	152.6	151.0 (minor)<br>150.9 (major)	152.2	150.9 (major)<br>151.1(minor)	151.0 (major)<br>151.5 (minor)	151.8
C4	99.5 (major)<br>100.4 (minor)	103.5	100.0 (major)<br>99.3 (minor)	96.9	100.0 (major)<br>99.3 (minor)	99.7 (major)<br>99.3 (minor)	97.9
C5	142.6 (major)<br>140.3 (minor)	144.0	142.3 (major)<br>142.9 (minor)	144.0	142.2 (major)<br>142.8 (minor)	143.0 (major)<br>143.1 (minor)	143.1
C6	112.7 (major)<br>118.4 (minor)	113.5	114.9 [a]	117.9	114.0 (major)<br>119.7 (minor)	114.5 (major)<br>120.2 (minor)	125.6
C7	130.1	129.0	122.0 [a]	122.0	128.1 (major)<br>128.5 (minor)	129.0(major)<br>129.3(minor)	130.4
C1′	128.1	129.0	122.1 [a]	117.9	129.0 (major)<br>129.3 (minor)	128.0 (major)<br>129.3 (minor)	131.6
C2′	109.5	112.3	160.5<br>^1J_F = 247.3	161.4/158.8<br>^1J_F ~ 262	113.4<br>^2J_F = 18.6	113.4<br>^2J_F = 18.5	109.1
C3′	147.9	148.3	102.8<br>^2J_F = 24.3	108.0	151.2<br>^1J_F = 240.8	152.1<br>^1J_F = 241.8	156.9/154.6<br>^1J_F ~ 247
R_3	55.6 (major)<br>55.5 (minor)	54.0	---	---	---	---	---
C4′	147.1 (major)<br>146.6 (minor)	148.3	158.7	156.2	145.0 (major)<br>^2J_F = 12.3<br>144.5 (minor)<br>^2J_F = 12.7	146.7 (major)<br>^2J_F = 11.8<br>146.3 (minor)	144.9
C5′	115.6 (major)<br>115. 3 (minor)	116.0	112.3	113.6	117.9 (major)<br>119.7 (minor)	118.0<br>^3J_F = 3.8	114.7
C6′	120.2 (major)<br>122.1 (minor)	119.5	128.2	128.5	123.4 (major)<br>123.1 (minor)	122.7 (major)<br>122.4 (minor)	122.9
C*i*	133.6 (major)<br>132.0 (minor)	133.2	133.6	130.2	133.6 (major)<br>129.0 (minor)	135.2 (major)<br>130.8 (minor)	132.3
C*o*	125.1 (major)<br>125.0 (minor)	126.4	125.1	124.3	125.1	125.4 (major)<br>125.5 (minor)	126.3
C*m*	128.7	129.0	128.7	126.5	128.6 (major)<br>128.8 (minor)	128.5 (major)<br>128.7 (minor)	128.1
C*p*	127.5	129.0	127.4	126.5	127.4 (major)<br>128.5 (minor)	127.1 (major)<br>127.7 (minor)	128.1
N1	[b]	−181.5	[b]	−177.2	−177.3 (major)<br>−179.7 (minor)	−177.0 (major)<br>−179.3 (minor)	−168.5
N2	[b]	−105.3	[b]	−111.9	[b]	[b]	−120.0

[a] Broad signal; [b] Not detected.

Table S2. *Cont.*

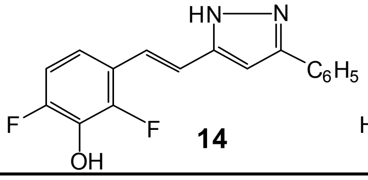

14

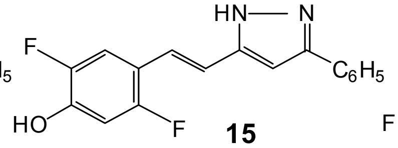

15

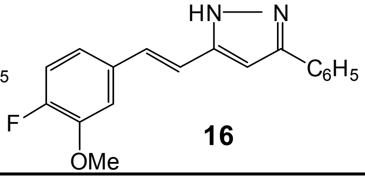

16

Nuclei	14		15		16				
**Solvent**	**DMSO-d_6**	**CPMAS**	**DMSO-d_6**	**CPMAS**	**$CDCl_3$**	**DMSO-d_6**	**DMSO-d_6**	**HMPA-d_{18}**	**CPMAS**
**(Temp)**	**295 K**	**300 K**	**295 K**	**300 K**	**300 K**	**360 K**	**295 K**	**295 K**	**300 K**
C3	151.0 (major)<br>153.3 (minor)	153.7	151.0	148.9	148.5	--	150.9	151.0 (major)<br>151.3 (minor)	152.1
C4	100.7 (major)<br>99.9 (minor)	97.1	100.6 [a] (major)<br>99.6 [a] (minor)	98.4	100.2<br>$^{1}J_{H} = 174.9$<br>$^{3}J_{H} = 3.4$	99.6	100.3 (major)<br>99.4 (minor)	100.3 (major)<br>99.4 (minor)	102.7
C5	141.8 (major)<br>143.0 (minor)	142.5	143.0 (minor)<br>142.0 (major)	143.9	147.2	--	142.0 (major)<br>142.9 (minor)	142.6 (major)<br>143.2 (minor)	142.1
C6	117.8	121.7	116.2 (major)<br>120.1 (minor)	116.8	117.8<br>$^{1}J_{H} \sim 158.3$	119.0	115.9 (major)<br>119.4 (minor)	116.1<br>122.5	115.0
C7	123.6	123.6	120.8 (major)<br>122.0 (minor)	124.9/123.7	129.9<br>$^{5}J_{F} = 1.6$<br>$^{1}J_{H} \sim 155.3$	128.2	128.9 (major)<br>128.3 (minor)	128.8 (major)<br>128.0 (minor)	130.5
C1′	121.3 (major)<br>120.7 (minor)	121.7	114.9 (major)<br>115.3 (minor)	119.8	133.3<br>$^{4}J_{F} = 3.8$	---	129.0	134.8 (major)	133.6
C2′	150.2<br>$^{1}J_{F} = 245.6$<br>$^{3}J_{F} = 5.8$	150.1	155.7<br>$^{1}J_{F} = 244.6$	157.5/154.8 [1+]<br>$J_{F} \sim 272$	111.0<br>$^{3}J_{F} = 1.8$<br>$^{1}J_{H} = 157.1$	111.8	110.9	111.6	113.8
C3′	134.0<br>$^{2}J_{F} = 16.1$<br>$^{2}J_{F} = 16.1$	153.1	105.0<br>$^{2}J_{F} = 26.6$	109.8	147.8<br>$^{2}J_{F} = 11.2$	---	147.4<br>$^{2}J_{F} = 10.9$	148.1<br>$^{2}J_{F} = 10.7$	148.2
R_3	---	---	---	---	56.2<br>$^{1}J_{H} = 144.7$	56.1	56.0	56.3	56.7
C4′	151.7<br>$^{1}J_{F} = 240.6$	135.5	145.7	148.0	152.4<br>$^{1}J_{F} = 249.2$<br>$^{3}J_{H} = 10.7$<br>$^{3}J_{H} = 8.5$<br>$^{2}J_{H} = 5.0$	---	151.3 (major)<br>$^{1}J_{F} = 249$<br>151.1 (minor)<br>$^{1}J_{F} = 246$	151.9 (major)<br>$^{1}J_{F} = 244$<br>151.7 (minor)<br>$^{1}J_{F} = 242$	152.1
C5′	111.9<br>$^{2}J_{F} = 18.6$	111.2	148.1<br>$^{1}J_{F} = 236.9$	153.5/150.9 [1]<br>$J_{F} \sim 269$	116.2<br>$^{2}J_{F} = 18.8$ [1]<br>$J_{H} = 162.6$	115.6	115.9 (major)<br>$^{2}J_{F} = 18$<br>116.0 (minor)<br>$^{2}J_{F} = 23$	116.6 (major)<br>$^{2}J_{F} = 22$<br>116.5 (minor)<br>$^{2}J_{F} = 24$	114.2
C6′	115.9<br>$^{3}J_{F} = 8.2$<br>$^{3}J_{F} = 3.6$	119.4	113.4<br>$^{2}J_{F} = 21.1$<br>$^{3}J_{F} = 5.4$	110.6	119.5<br>$^{3}J_{F} = 6.8$<br>$^{1}J_{H} = 161.6$	121.7	119.4 (major)<br>121.5 (minor)	119.2<br>$^{3}J_{F} = 6.6$	118.4
C*i*	133.5	126.8	133.5	129.3	131.1	---	133.5 (major)<br>134.1 (minor)	135.1 (major)<br>135.2 (minor)	133.6

Table S2. *Cont.*

Nuclei	14		15		16				
C*o*	125.1	126.8	125.1	128.1	125.6<br>$^{1}J_{H}$ = 158.4<br>$^{3}J_{H}$ = 7.1<br>$^{3}J_{H}$ = 7.1	124.8	125.1	125.3 (major)<br>125.5 (minor)	124.3<br>127.2
C*m*	128.8	132.3	128.7	128.9	128.9<br>$^{1}J_{H}$ = 159.8<br>$^{3}J_{H}$ = 7.7	128.0	128.7 (major)<br>129.0 (minor)	128.6 (major)<br>128.8 (minor)	128.3
C*p*	127.6	129.0	127.5 (major)<br>128.3 (minor)	130.9	128.4<br>$^{1}J_{H}$ = 161.2<br>$^{3}J_{H}$ = 7.5<br>$^{3}J_{H}$ = 7.5	127.1	127.4 (major)<br>128.1 (minor)	127.1 (major)<br>127.7 (minor)	128.3
N1	−178.3	−191.7	−178.6	−152.2	[a]	[a]	−177.6 (major)<br>−179.2 (minor)	−176.4	−181.1
N2	[a]	−93.9	[a]	−141.1	[a]	[a]	[a]	[a]	−94.7

[a] Not detected.

ORTEP plot (40% Ellipsoid Probability) of 13 and 16 Showing the Labeling of Their Asymmetric Units

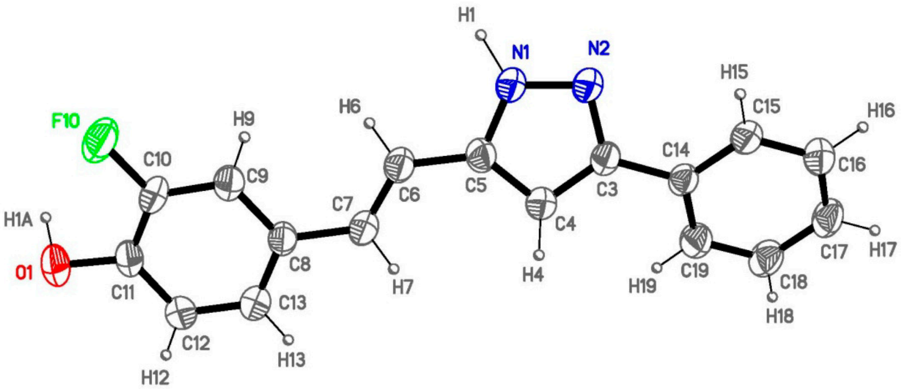

Figure S1. (*E*)-5-[β-(3-Fluoro-4-hydroxyphenyl)-ethenyl]-3-phenyl-1*H*-pyrazole (**13**).

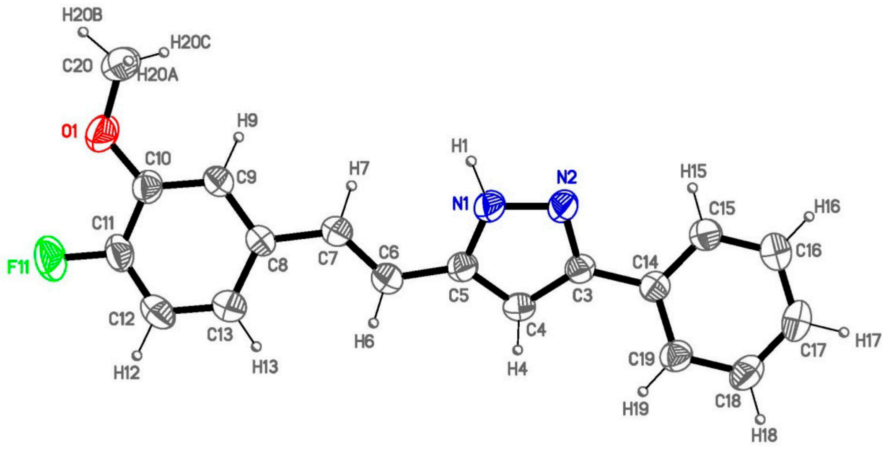

Figure S2. (*E*)-5-[β-(4-Fluoro-3-methoxyphenyl)-ethenyl]-3-phenyl-1*H*-pyrazole (**16**).

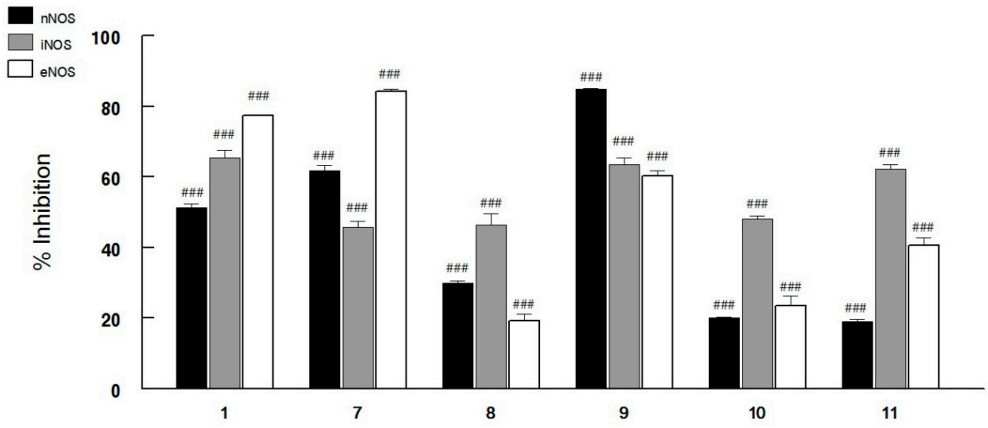

Figure S3. Percentage of inhibition of nNOS, iNOS and eNOS activities in the presence of the tested curcuminoid pyrazoles (**1**, **7–11**) compared to control (0% inhibition). Experimental data represent the means ± S.E.M. of three independent experiments (n = 3), each one performed in triplicate $^{###}p < 0.001$ *vs.* control.

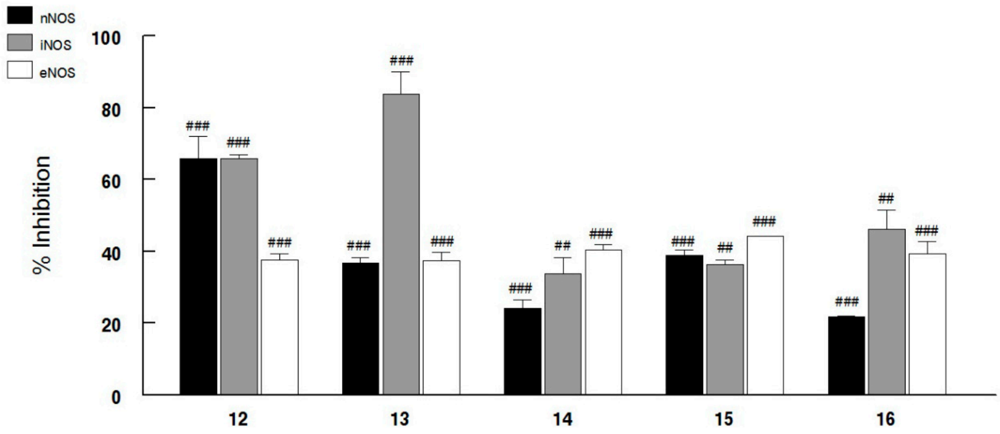

Figure S4. Percentage of inhibition of nNOS, iNOS and eNOS activities in the presence of the tested curcuminoid pyrazoles (**12–16**) compared to control (0% inhibition). Experimental data represent the means ± S.E.M. of three independent experiments (n = 3), each one performed in triplicate $^{##}p < 0.01$ $^{###}p < 0.001$ *vs.* control.

Table S3. Free Wilson matrix used to obtain the statistical results of Table 5.

Compound	F2′	F3′	F4′	F5′	OH-3′	nNOS	iNOS	eNOS
**1**	0	0	0	0	0	2.560	3.265	3.870
**7**	0	0	0	0	0	3.085	2.280	4.205
**8**	0	0	0	0	0	1.495	2.320	0.960
**9**	0	0	0	0	0	4.245	3.175	3.015
**10**	0	0	0	0	0	1.000	2.400	1.170
**11**	0	0	0	0	0	0.950	3.105	2.030
**12**	1	0	0	0	0	65.900	65.800	37.600
**13**	0	1	0	0	0	36.800	83.700	37.400
**14**	1	0	1	0	1	24.100	33.600	40.300
**15**	1	0	0	1	0	38.900	36.300	44.200
**16**	0	0	1	0	0	21.700	46.000	39.300

nNOS = (65.9 ± 2.5) F2′ + (36.8 ± 2.5) F3′ + (21.7 ± 2.5) F4′ − (27.0 ± 3.6) F4′ − (63.5 ± 4.4) OH-3′, n = 11, R^2 = 0.995;

iNOS = (65.8 ± 2.8) F2′ + (83.7 ± 2.8) F3′ + (46.0 ± 2.8) F4′ − (29.5 ± 3.9) F4′ − (78.2 ± 4.8) OH-3′, n = 11, R^2 = 0.997;

nNOS = (37.6 ± 2.9) F2′ + (37.4 ± 2.9) F3′ + (39.3 ± 2.9) F4′ + (6.6 ± 3.0) F4′ − (36.6 ± 4.9) OH-3′, n = 11, R^2 = 0.994.
